# Behavioural and service-context correlates of tetanus vaccination intention after trauma exposure in China: a cross-sectional BeSD study

**DOI:** 10.3389/fpubh.2026.1941921

**Published:** 2026-09-21

**Authors:** Quanjun Fang, Jilai Mao, Xiaoying Gong, Xinyuan Liu, Canjie Zheng, Guoping Cao, Zhiying Yin

**Affiliations:** 1 Department of Immunity, Quzhou Center for Disease Control and Prevention, Quzhou, Zhejiang, China; 2 Department of Immunity, Jiangshan Center for Disease Control and Prevention, Jiangshan, Zhejiang, China

**Keywords:** BeSD, immunisation history, post-exposure prophylaxis, structural equation modelling, tetanus, trauma, vaccination intention

## Abstract

**Introduction:**

Trauma-care encounters provide an opportunity to assess tetanus risk and deliver post-exposure prophylaxis. We primarily examined behavioural, social, and service-context factors associated with tetanus vaccination intention using the Behavioural and Social Drivers of Vaccination (BeSD) framework. We additionally described documented vaccination, guideline-based indications, and exploratory algorithm-classified guideline concordance among trauma-exposed adults.

**Methods:**

A cross-sectional study was conducted at 12 healthcare sites in Quzhou, China, from March to May 2026. Vaccination intention was assessed via a questionnaire, whereas documented active vaccination was verified from clinical and immunisation records. Guideline-based indications were determined from wound characteristics and previous immunisation history using a prespecified algorithm, and guideline concordance reflected whether documented management matched the algorithm-based recommendation. Logistic regression and structural equation modelling were used to assess factors associated with vaccination intention.

**Results:**

Among 657 participants, 341 (51.9%) reported a willingness to receive tetanus vaccination. Animal-related injury, thinking and feeling, social processes, and practical accessibility were positively associated with willingness, with practical accessibility showing the strongest association with latent intention. Overall, 642 participants had a guideline-based indication for active vaccination, while 126/657 (19.2%) had documented active vaccination within 7 days. Among 333 participants who were both willing and guideline-indicated, 100/333 (30.0%) had documented vaccination. Separately, 119/657 (18.1%) met the exploratory algorithm-classified guideline-concordance definition.

**Conclusion:**

BeSD-related perceptions, particularly perceived practical accessibility, were associated with tetanus vaccination intention during the trauma-care episode. However, documented vaccination and guideline-concordant prophylaxis could not be fully interpreted because reliable assessment depended on complete immunisation histories and wound documentation. Because the temporal sequence between questionnaire completion and clinical care could not be verified for all participants, the findings should be interpreted as cross-sectional associations. Algorithm-classified guideline concordance should be viewed as an exploratory, data-dependent classification rather than a direct measure of clinician adherence to prophylaxis guidelines. These findings highlight the potential value of improving immunisation record verification and facilitating accessible vaccination services, while further studies with more complete clinical documentation are needed to evaluate guideline-concordant prophylaxis.

## 1 Introduction

Tetanus is uncommon but remains clinically important because it is preventable and potentially fatal. Trauma-care encounters provide clinicians with a narrow window to assess wound risk, verify tetanus vaccination status, and provide active or passive immunisation when indicated (1, 2). Guideline-concordant prophylaxis depends on the combination of wound characteristics, previous vaccination, and appropriate immunological intervention. In practice, incomplete records and uncertain adult immunisation histories often complicate this assessment, especially in emergency and outpatient trauma care (3, 4).

In China, stand-alone tetanus vaccination for trauma-related prevention has not been incorporated into a routine universal adult booster programme and is mainly delivered through trauma-related care or designated vaccination services. Emergency departments, animal-injury clinics, vaccination services, and wound-care clinics therefore become the main points at which prevention decisions are made (5, 6). Patients may be asked to decide about vaccination while receiving wound treatment. Clinicians must often make recommendations with limited information about previous immunisation. This service context can create gaps between clinical indication, stated willingness, and documented vaccine receipt.

Clinical work on post-traumatic tetanus prevention has rightly focused on algorithms, vaccine availability, and adherence to prophylaxis recommendations (3, 5). Those topics remain central, but they leave a patient-facing question unresolved. Why does one injured adult accept or complete prophylaxis during the visit while another does not? In the care room, that decision may depend on perceived disease risk, confidence in the vaccine, advice from clinicians and family, service access, cost, and trust in the health system (7, 8).

The Behavioural and Social Drivers of Vaccination (BeSD) framework is useful here because it treats vaccination as more than an attitude or knowledge outcome (9). Within the BeSD framework, vaccination-related considerations are organised into thinking and feeling, social processes, and practical issues (9). Researchers have used BeSD-based tools to study vaccine intention and uptake in other populations, but trauma-exposed adults remain under-studied. It is also unclear whether BeSD constructs relate only to stated intention or whether they track documented vaccination and algorithm-classified guideline concordance in post-exposure prophylaxis (9, 10).

Animal-related injuries often present through rabies post-exposure prophylaxis clinics, where wound management, immunisation-history review, and post-exposure decisions may be organised within a dedicated care pathway (11). In contrast, non-animal-related injuries in this study were managed across emergency, surgical outpatient, and primary wound-care settings. This contrast provided a rationale for comparing vaccination intention and documented clinical management across trauma-care contexts.

We primarily investigated behavioural, social, and service-context factors associated with tetanus vaccination intention among trauma-exposed adults in Quzhou, China, using the Behavioural and Social Drivers of Vaccination (BeSD) framework. We developed and evaluated a BeSD-based questionnaire and examined associations between BeSD constructs and vaccination intention using logistic regression and structural equation modelling. We additionally described documented vaccination within 7 days, exploratory algorithm-classified guideline concordance, and the gap between stated willingness and documented vaccination. By integrating behavioural measures with post-exposure clinical records, this study aimed to identify potential targets for improving adult tetanus prevention after trauma. Documented vaccination and guideline-classified prophylaxis were evaluated as secondary clinical outcomes and were not included as downstream variables in the BeSD conceptual model.

## 2 Methods

### 2.1 Study design and setting

This cross-sectional study was conducted among trauma-exposed adults seeking care at 12 participating healthcare sites in Quzhou, Zhejiang Province, China, between March and May 2026. Quzhou has a multi-level healthcare delivery system consisting of county-level or higher-level hospitals, community health service centres, township hospitals, and other specialised outpatient facilities. Among the eligible trauma-care facilities identified before the study’s initiation, 12 sites were selected to represent different levels of healthcare delivery and major trauma-care pathways. The participating sites represented three institutional categories: county-level or higher-level hospitals, community health service centres, and township hospitals. Within these institutions, participants were recruited from four clinical settings: emergency departments, surgical outpatient clinics, animal-injury clinics, and primary vaccination or wound-care clinics. These settings were included to capture the heterogeneity of trauma-care pathways in the study area. Guided by the BeSD framework, we assessed vaccination intention, documented active vaccination, and exploratory algorithm-classified guideline concordance among trauma-exposed adults.

The study protocol received ethical approval from the Ethics Committee of Quzhou Centre for Disease Control and Prevention (approval no. 2026013). All participants provided written informed consent before completing the questionnaire. Questionnaire, clinical record, and immunisation record data were linked using unique study identifiers and analysed in de-identified form, and the study was reported in accordance with the STROBE guidelines for observational studies (12).

### 2.2 Participants

Patients were screened for eligibility at the participating trauma-care settings. Participants were eligible if they were aged ≥18 years, presented for medical care following trauma exposure, and were able to complete the questionnaire independently or with assistance from investigators. Participants were excluded if they were aged <18 years, did not have a trauma-related healthcare encounter, were unable to complete the questionnaire due to severe injury, impaired consciousness, cognitive or behavioural conditions, or communication difficulties, or declined participation. Contraindications to tetanus-containing vaccination or previous severe vaccine allergy were considered only for clinical prophylaxis-related analyses and were not exclusion criteria for the questionnaire-based intention analyses. Written informed consent was required for enrolment but was not used to define screening eligibility. Individuals who met the eligibility criteria but declined participation or did not complete recruitment were recorded as eligible non-participants.

A multi-stage stratified site-selection approach followed by consecutive patient recruitment was used. Healthcare facilities providing trauma-related care or tetanus post-exposure management in Quzhou were identified as the sampling frame. Twelve healthcare sites were selected to represent different levels of healthcare delivery, including county-level or higher-level hospitals, community health service centres, and township hospitals. Within selected sites, relevant trauma-care settings, including emergency departments, surgical outpatient clinics, animal-injury clinics, and vaccination or wound-care clinics, were included. Consecutive eligible trauma-exposed adults presenting during the study period were invited to participate. The site-selection strategy aimed to capture heterogeneity across trauma-care environments rather than to produce a population-weighted sample representative of all trauma-related healthcare encounters in Quzhou. Recruitment differed by trauma type because animal-related injuries were more frequently managed through animal-injury clinics, whereas non-animal-related injuries were mainly recruited from emergency departments and surgical outpatient clinics (Supplementary Table S1). Site-specific screening, eligibility, participation, quality-control exclusions, and the distribution of animal-related and non-animal-related injuries are reported in Supplementary Table S1.

The sample size was calculated using a single-proportion formula, assuming an expected willingness to vaccinate of 45% based on previous findings among trauma patients in China (6). A two-sided alpha of 0.05 and a 5% margin of error were applied. After applying a design effect of 1.5 and allowing for 10% invalid responses, at least 640 valid questionnaires were required. The achieved sample was also considered adequate for the planned SEM based on published recommendations regarding the ratio of observations to estimated parameters (13).

### 2.3 Clinical indication adjudication and immunisation record verification

Participants were intended to complete the questionnaire before clinical consultation and prophylaxis decisions. However, questionnaire completion was not systematically timestamped relative to physician assessment, clinical wound assessment, healthcare provider recommendation, or vaccination administration. Therefore, the individual-level temporal sequence among these components of the trauma-care episode could not be reliably reconstructed. Wound characteristics used for analytical classification were abstracted from source medical records rather than from questionnaire self-report; however, the timing of the underlying clinical wound assessment relative to questionnaire completion was not consistently documented. Accordingly, questionnaire-based BeSD measures were interpreted as care episode perceptions rather than confirmed pre-consultation exposures. To assess the reproducibility of wound classification, a stratified random sample of 197 source records was selected, stratified by participating institution and the initially recorded wound category, and these were independently reviewed by two trained emergency physicians. Reviewers recorded prespecified features, including wound depth, puncture or penetration, bite or saliva exposure, contamination, retained foreign material, tissue viability, crush injury, open fracture, burn or frostbite, time from injury to presentation, and documented wound management. Time from injury to presentation and documented wound management were abstracted for descriptive and adjudication purposes but were not used as standalone wound classification criteria. Wound management information contributed to classification only when it documented a prespecified severe or complex feature, such as extensive debridement or surgical exploration. Each feature was coded as present, absent, or undocumented. Reviewers were blinded to questionnaire responses, stated vaccination intention, and documented active or passive immunisation. Wounds were then classified according to the predefined operational criteria in Supplementary Table S2. Severe or complex features took priority over contaminated or deep features. A wound was classified as clean and minor only when the source record explicitly described a superficial clean minor wound and contained sufficient information to exclude puncture or penetration, bite or saliva exposure, contamination, devitalised tissue, crush injury, open fracture, burn, frostbite, or other complex features. Records lacking sufficient information or containing conflicting information were classified as indeterminate. Disagreements were resolved by a third senior clinician.

Previous tetanus immunisation history was classified into three categories. Formally verified previous vaccination required formal records documenting primary series status and, where applicable, the date of the most recent tetanus-containing vaccine dose. When formal records were unavailable, participants who provided a definite report of no previous tetanus vaccination or a most recent dose more than 10 years previously were retained in a separate self-reported inadequate-history category. Participants who could not provide a definite history were classified as having unknown or uncertain immunisation history. Neither the self-reported inadequate-history category nor the unknown or uncertain category was considered evidence of verified protection for the clinical prophylaxis algorithm. Unknown or uncertain immunisation history was retained as a separate analytic category rather than treated as missing data and was not imputed.

A guideline-based indication for active tetanus vaccination was determined according to wound category, verified completion of the primary tetanus-containing vaccination series, and the interval since the most recent documented tetanus-containing vaccine dose (1, 2). Participants with clean minor wounds, a verified completed primary series, and a documented tetanus-containing vaccine dose within the preceding 10 years were classified as having no indication for active vaccination. Participants with contaminated, deep, severely contaminated, or complex wounds, a verified completed primary series, and a documented dose within the preceding 5 years were likewise classified as having no indication for an additional active dose. Participants without verifiable evidence of completed primary-series status and an interval-appropriate dose were not classified as having adequate protection for this clinical algorithm.

Fifteen participants were classified as having no guideline-based indication for active tetanus vaccination after adjudication of wound category and immunisation history. These participants were retained in the overall analytic cohort because they had completed valid questionnaires and remained relevant to analyses of baseline vaccination intention, BeSD constructs, documented clinical management, and algorithm-classified guideline concordance. They were excluded only from the denominator of the intention–vaccination gap analysis. The remaining 642 participants constituted the clinically indicated subgroup for analyses comparing stated willingness with documented active vaccination.

### 2.4 Questionnaire development and pilot testing

The questionnaire comprised two sections. The first collected demographic, clinical, and healthcare-related characteristics, including participant demographics, recruitment setting, trauma characteristics, wound severity, previous tetanus immunisation history, and post-exposure prophylaxis received during the current episode of care.

The second section consisted of a BeSD-based tetanus vaccination intention questionnaire. Items were developed using the WHO BeSD framework, literature evidence, local tetanus prophylaxis practices, and expert input (1, 2, 14). A multidisciplinary expert panel reviewed the draft for content relevance, wording clarity, local applicability, and consistency with tetanus post-exposure practice. The panel included expertise in immunisation programme delivery, emergency or surgical care, epidemiology, and behavioural or public health research. Items were revised or removed when they were ambiguous, redundant, difficult to answer in a trauma-care encounter, or poorly aligned with the intended BeSD construct. The revised questionnaire was pilot tested among 50 trauma-exposed adults to assess clarity, feasibility, completion time, and preliminary reliability. Final psychometric performance was then evaluated in the full study sample.

The final measurement model was specified on theoretical grounds as four latent constructs: thinking and feeling, social processes, practical accessibility, and vaccination intention. Item assignment was guided primarily by the WHO BeSD conceptual framework and the intended content of each item, with wording and construct alignment further refined through expert review and pilot testing. Thus, the final factor structure was theory-driven rather than selected solely on the basis of sample-specific model-fit statistics. Its empirical adequacy was subsequently evaluated by confirmatory factor analysis in the full analytic sample.

The four constructs captured individual perceptions (severity, susceptibility, vaccine confidence, benefits and adverse-event concerns), social influences (family and peer support, healthcare-provider recommendation and social norms), perceived service accessibility during the current care episode (physical access, time and process convenience, affordability, and trust in service delivery), and willingness to vaccinate. These domains encompassed individual perceptions (severity, susceptibility, vaccine confidence, benefits and adverse-event concerns), social influences (family and peer support, healthcare provider recommendation and social norms), perceived service accessibility during the current care episode (physical access, time and process convenience, affordability, and trust in service delivery), and willingness to vaccinate. These measures were interpreted as care-episode perceptions and correlates of vaccination intention and were not treated as confirmed pre-consultation exposures or as variables with established temporal precedence. Healthcare-provider recommendation (SP3) was retained within the social-processes construct because the WHO BeSD framework places health-worker recommendation within the social-processes domain, reflecting the interpersonal context in which vaccination decisions occur. However, in an acute trauma-care encounter, this item may also capture aspects of healthcare delivery. Therefore, a sensitivity analysis excluding SP3 was conducted to examine the robustness of the measurement model. Affordability was assessed using the local out-of-pocket cost of 203 yuan per dose (180 yuan for vaccine purchase and 23 yuan for the vaccination service fee). Vaccination intention was measured by three items assessing willingness to consider, attempt, and invest resources in vaccination.

BeSD items were scored on a 5-point Likert scale (1–5). Reverse-coded items were transformed before analysis. All items were oriented such that higher scores represented more favourable perceptions, greater accessibility, and stronger vaccination intention. Construct scores were calculated as the mean of the corresponding items.

### 2.5 Outcomes

The primary outcome for descriptive and logistic regression analyses was binary tetanus vaccination intention following the current trauma exposure. Binary intention was derived from a single global 5-point item asking whether the participant was willing to receive a tetanus-containing vaccine following trauma exposure. Responses of “willing” or “very willing” were classified as affirmative willingness, whereas “uncertain,” “unwilling,” and “very unwilling” were classified as not willing/uncertain. Participants who reported being unwilling or uncertain were combined into a single reference category because neither response represented affirmative willingness at the trauma-care encounter. The global binary outcome was used for the conventional regression analysis because it provided a directly interpretable distinction between affirmative willingness and the absence of affirmative willingness during the care episode and was consistent with the operational definition subsequently used to examine the intention-vaccination gap.

For confirmatory factor analysis and structural equation modelling, vaccination intention was modelled as a latent construct measured by three items: INT1, willingness to consider vaccination; INT2, willingness to try to receive vaccination; and INT3, willingness to invest time and money in vaccination. This three-item measure captured gradations in the strength and behavioural commitment of vaccination intention and was therefore more appropriate for the latent-variable measurement and structural models. The binary and latent intention outcomes were complementary rather than interchangeable: logistic regression evaluated factors associated with affirmative willingness, whereas structural equation modelling assessed theory-consistent associations among the latent BeSD constructs and latent vaccination intention. To determine whether the regression findings depended on this operationalisation of intention, we additionally examined the mean of INT1-INT3 as an alternative continuous outcome in a sensitivity analysis.

Secondary behavioural and clinical outcomes included documented receipt of a tetanus-containing vaccine within 7 days after the index trauma-care encounter, receipt of passive immunisation, exploratory algorithm-classified guideline concordance, and consistency between stated willingness and documented active vaccination. The 7-day interval was used as a pragmatic operational window to capture early vaccination temporally related to the index trauma-care encounter rather than as a guideline-mandated clinical cut-off. Active vaccination was ascertained through post-encounter review of source clinical records and the Zhejiang provincial immunisation information system rather than through prospective participant contact, while passive immunisation was verified through source clinical records. Vaccinations administered within Quzhou are routinely recorded in the provincial immunisation information system, allowing vaccinations received at healthcare facilities other than the index recruitment setting to be identified. Participant self-report alone was not considered sufficient evidence of documented vaccination. Vaccination ascertainment was based on record review rather than prospective participant contact. For participants without documented vaccination within 7 days, immunisation records were additionally searched until 30 June 2026 to identify any subsequent tetanus-containing vaccination. Passive immunisation referred to documented administration of tetanus immunoglobulin or tetanus antitoxin during the observed episode of care.

A guideline-based indication for active tetanus vaccination was determined from the combination of wound category, verified completion of the primary tetanus-containing vaccination series, and the interval since the most recent documented tetanus-containing vaccine dose, according to the prespecified wound- and immunisation-history-based algorithm (1, 2). Active vaccination was considered not indicated for participants with clean minor wounds who had a verified completed primary series and a documented tetanus-containing vaccine dose within the preceding 10 years. Active vaccination was also deemed not indicated for participants with contaminated, deep, severely contaminated, or complex wounds who had a verified completed primary series and a documented tetanus-containing vaccine dose within the preceding 5 years. Participants with incomplete, outdated, or unverifiable protection were classified as requiring active vaccination according to the prespecified algorithm. The requirement for passive immunisation was additionally determined by wound category and whether completion of the primary vaccination series could be verified.

Completed primary vaccination was defined as documented receipt of at least three doses of a tetanus-containing vaccine or verified completion of the age-appropriate childhood immunisation schedule. In the absence of formal records, a definite self-report of no vaccination or a most recent dose more than 10 years previously was retained as a self-reported inadequate-history category; histories without a definite report were classified as unknown or uncertain.

Early documented active vaccination within 7 days by stated intention was evaluated exclusively in the clinically indicated subgroup. The numerator was the number of participants who expressed affirmative willingness and had no documented receipt of a tetanus-containing vaccine within 7 days. The denominator was participants who expressed affirmative willingness and for whom active tetanus vaccination was indicated according to the prespecified clinical algorithm. Participants without an indication for active vaccination were excluded from both the numerator and denominator because non-vaccination represented appropriate guideline-concordant management for these participants.

The intention-vaccination gap was distinguished from the algorithm-classified guideline-concordance outcome. The former described the transition from stated willingness to documented active vaccination among clinically eligible participants, whereas the latter used the prespecified study algorithm to evaluate whether the documented combination of active vaccination, passive immunisation, or non-treatment matched the guideline-based recommendation derived from the available wound and immunisation-history information for each participant.

Algorithm-classified guideline concordance was evaluated retrospectively in the overall analytic cohort using the prespecified study algorithm and incorporated both indicated treatment and appropriate non-treatment. The classification algorithm is provided in Supplementary Table S3 and the operational criteria for wound-category adjudication in Supplementary Table S2. The primary cohort analysis used the wound category recorded or derived during original data collection, together with documented active and passive interventions. Histories lacking formal verification were classified as either self-reported inadequate history or unknown or uncertain history, according to whether the participant provided a definite account of no previous vaccination or a most recent dose more than 10 years previously. Neither category was treated as evidence of verified protection. Participants for whom neither active nor passive immunisation was indicated were classified by the study algorithm as guideline concordant when neither intervention was documented. The duplicate-adjudication substudy did not replace the primary wound classification for the full cohort. Records that remained indeterminate after duplicate review were excluded only from the adjudication-based sensitivity analysis. Because the outcome depended on routine record detail and incomplete immunisation-history verification was common, it was treated as an exploratory algorithm-classified outcome rather than as a direct measure of actual clinician adherence to prophylaxis guidelines or service quality.

### 2.6 Data collection and quality control

Before study initiation, investigators underwent standardised training on participant screening, informed consent procedures, questionnaire administration, trauma and wound classification, and extraction of post-exposure prophylaxis information. Eligible participants were consecutively recruited at each survey site during the study period. Site-level screening and recruitment logs were maintained to record the numbers screened, eligible, declining participation, not completing recruitment for other reasons, completing the questionnaire, and subsequently excluded during quality control. Participants completed the questionnaire as soon as practicable after registration or triage. Investigators administering the questionnaire were not involved in clinical decision-making and did not provide vaccine-related information. Nevertheless, because questionnaire completion was not systematically timestamped against physician assessment, wound assessment, provider recommendation, or vaccination administration, the temporal order could not be established for every participant. Questionnaire responses were therefore analysed as care-episode perceptions rather than confirmed antecedent exposures.

Questionnaires were checked immediately after completion for completeness and logical consistency. Ten percent of questionnaires were randomly selected each day for quality control. Paper-based data underwent double entry and verification, while electronic data were screened for duplicates, implausible completion times, patterned responses, missing key variables, and logical errors. Invalid records were removed before analysis. The overall STROBE participant flow is shown in Supplementary Figure S1, and site-specific screening and recruitment data are presented in Supplementary Table S1.

### 2.7 Statistical analysis

Categorical variables were summarised as frequencies and percentages. Continuous variables and ordinal scale scores derived from the BeSD questionnaire were summarised using means and standard deviations when approximately normally distributed, or medians and interquartile ranges when distributional assumptions were not met. Group differences in categorical demographic and clinical characteristics were assessed using chi-square tests or Fisher’s exact tests. Differences in continuous BeSD construct scores between two groups were assessed using independent-samples t-tests when appropriate, or Mann–Whitney U tests when distributional assumptions were not met. For comparisons involving more than two groups, one-way analysis of variance (ANOVA) or Kruskal–Wallis tests were used as appropriate. To assess whether recruitment composition differed by trauma type, the distribution of participants across recruitment settings was compared between animal-related and non-animal-related injuries using a chi-square test, with Cramér’s V reported as a measure of effect size.

Hierarchical multivariable logistic regression was performed to identify factors associated with binary vaccination intention. Model 1 adjusted for sociodemographic characteristics, including sex, age group, education, monthly income, and residence. Model 2 additionally incorporated trauma-related and healthcare-related factors, including trauma type, wound category, recruitment institution, recruitment setting, and previous tetanus immunisation history. The latter was categorised into three groups: formally verified previous vaccination, self-reported no vaccination or most recent dose more than 10 years previously, and unknown or uncertain history. Model 3 further incorporated the three BeSD construct scores: thinking and feeling, social processes, and practical accessibility. Results from multivariable logistic regression models were reported as adjusted odds ratios (aORs), 95% confidence intervals (CIs), and two-sided *p* values. Crude odds ratios (ORs) were reported only for unadjusted comparisons, whereas adjusted odds ratios (aORs) were used for multivariable regression models. For categorical predictors, the reference categories were male sex, age 18–29 years, junior middle school or below, monthly income <3,000 yuan, rural residence, non-animal-related injury, clean minor wound, primary institution, emergency department, and formally verified previous tetanus immunisation, as applicable to each model. For the continuous BeSD construct scores, aORs represented the change in odds associated with a one-point increase in the corresponding 1–5 mean score. Multicollinearity was evaluated using variance inflation factors (VIFs). VIF values were interpreted based on established methodological recommendations, with values <5 considered indicative of acceptable collinearity (15, 16). Because participants were nested within 12 healthcare sites, potential intra-site correlation was assessed in a sensitivity analysis. The fully adjusted logistic regression model (Model 3) was re-estimated using cluster-robust standard errors at the healthcare site level, with the 12 participating healthcare sites treated as clusters and the same covariate specification retained. A separate sensitivity analysis repeated Model 3 after excluding the healthcare-provider recommendation item from the social processes score. As an outcome-operationalisation sensitivity analysis, the fully adjusted Model 3 was additionally re-estimated using the mean of INT1–INT3 as an alternative continuous vaccination intention outcome. Multivariable linear regression was used with the same sociodemographic, trauma-related, healthcare-related, and BeSD variables as in the primary Model 3. Unstandardised regression coefficients (B) with 95% CIs, standardised β coefficients, and *p* values were reported. This analysis assessed whether the substantive findings were robust to using a graded three-item measure of intention rather than the binary global outcome. Because only 12 healthcare site clusters were available, cluster-robust inference was interpreted as supportive rather than definitive.

The measurement model was evaluated before SEM. Internal consistency was assessed using both Cronbach’s α and McDonald’s ω (17–19). Convergent validity was evaluated using standardised factor loadings, composite reliability (CR), and average variance extracted (AVE), whereas discriminant validity was assessed using the heterotrait-to-monotrait ratio (HTMT) (18, 20). Values of α, ω, and CR ≥ 0.70 and AVE ≥ 0.50 were used as conventional reference benchmarks, and HTMT values <0.85 were considered supportive of discriminant validity (18–20).

Confirmatory factor analysis (CFA) was used to evaluate the prespecified four-factor measurement model (18, 21, 22). CFA and SEM were estimated using maximum likelihood. Because item distributions showed no severe deviation from approximate normality, with skewness values ranging from −0.30 to 0.13 and kurtosis values ranging from −1.05 to −0.72 across all Likert-type items (Table 1), the 5-point Likert-type items were treated as continuous indicators. Model fit was evaluated using the chi-square statistic (χ^2^), the χ^2^/df ratio, comparative fit index (CFI), Tucker–Lewis index (TLI), root mean square error of approximation (RMSEA) with its 90% confidence interval, and standardised root mean square residual (SRMR). Model fit indices were interpreted jointly rather than relying on a single threshold (18, 21, 22).

**Table 1:** Item-level descriptive statistics and standardised factor loadings from the four-factor confirmatory factor analysis (*n* = 657).

Construct	Item	Questionnaire item content	Mean	SD	Skewness	Kurtosis	Standardised loading
Thinking and feeling	TF1	Perceived severity of tetanus	3.18	1.21	−0.08	−0.89	0.74
	TF2	Perceived susceptibility after trauma exposure	3.05	1.19	−0.01	−0.89	0.66
	TF3	Confidence in vaccine effectiveness	3.31	1.22	−0.21	−0.91	0.77
	TF4	Confidence in vaccine safety	3.12	1.27	−0.08	−1.02	0.74
	TF5	Perceived benefit of vaccination	3.34	1.19	−0.23	−0.87	0.77
	TF6	Low concern about adverse reactions, reverse-coded	2.96	1.23	0.01	−0.95	0.64
Social processes	SP1	Family support	3.21	1.23	−0.17	−0.97	0.78
	SP2	Peer support	3.06	1.24	−0.1	−0.91	0.74
	SP3	Healthcare-provider recommendation	3.42	1.22	−0.3	−0.9	0.82
	SP4	Perceived social norm	3.09	1.21	−0.1	−0.95	0.71
Practical accessibility	PI1	Physical accessibility of vaccination service	3.30	1.22	−0.21	−0.9	0.77
	PI2	Time convenience	3.15	1.23	−0.13	−0.97	0.73
	PI3	Affordability of 203 yuan per dose	2.88	1.25	0.05	−1.05	0.75
	PI4	Process convenience	3.27	1.19	−0.19	−0.83	0.78
	PI5	Trust in service delivery	3.25	1.18	−0.26	−0.84	0.7
Vaccination intention	INT1	Would consider vaccination	3.08	1.21	−0.06	−0.91	0.83
	INT2	Would try to receive vaccination	2.96	1.21	0.06	−0.86	0.87
	INT3	Willing to invest time and money	2.91	1.24	0.13	−0.72	0.82

CFA was conducted in the full analytic cohort (*n* = 657).

After data quality screening, all 657 participants in the final analytic cohort had complete responses to all 18 BeSD items (657/657; item-level completeness, 100%). No participant had one or more missing BeSD items, and no item-level imputation was therefore performed. CFA and SEM were both conducted using the complete BeSD data from all 657 participants. Missing data handling was specified according to variable type. Questionnaires with missing key outcome or exposure data were excluded during data quality screening. In the final analytic cohort, complete data were available for binary vaccination intention, all 18 BeSD items, and the major sociodemographic, trauma-related, and healthcare-related covariates used in the primary models; therefore, no imputation was performed. Unknown or uncertain previous tetanus immunisation history was retained as a separate analytic category rather than treated as missing. For non-key covariates, complete-case analysis was used where applicable. Records remaining indeterminate after duplicate wound-category adjudication were excluded only from the corresponding sensitivity analysis. Common method bias was assessed using Harman’s single-factor test and a common latent factor sensitivity model (23).

SEM was used to examine theory-consistent associations among BeSD constructs and latent vaccination intention. Because the study used a cross-sectional design, SEM coefficients were interpreted as associations rather than causal effects. The hypothesised model specified directional paths from thinking and feeling to social processes, from social processes to practical accessibility, from thinking and feeling to practical accessibility, and from each BeSD construct to latent vaccination intention. Given the cross-sectional design and the unverified temporal sequence of questionnaire assessment and clinical care, these directional paths were used to represent theory-consistent statistical associations and were not interpreted as establishing temporal precedence or causal effects. Standardised coefficients, standard errors, *p* values, and 95% CIs were reported where available. Sensitivity analyses excluding SP3 were conducted for the measurement model, logistic regression model, and SEM to evaluate whether the classification of healthcare-provider recommendation within the social-processes construct materially influenced psychometric properties or substantive associations. A separate multivariable logistic regression examined the association between injury type and documented active vaccination within 7 days. Model A adjusted for sex, age group, education, monthly income, residence, wound category, recruitment institution, recruitment setting, and previous tetanus immunisation history. Model B additionally included thinking and feeling, social processes, and practical accessibility. Because temporal ordering within the care episode could not be established, Model B was interpreted as an exploratory context-adjusted model rather than a causal model. Participant characteristics, binary vaccination intention, BeSD measurement properties, logistic regression, CFA, SEM, and documented clinical management were analysed in the overall analytic cohort of 657 participants. Fifteen participants without a guideline-based indication for active tetanus vaccination were retained in this overall cohort and excluded only from analyses comparing stated willingness with documented active vaccination within 7 days. The remaining 642 participants formed the clinically indicated subgroup for that comparison. Denominators were reported explicitly for all secondary behavioural outcomes.

Secondary analyses described documented active vaccination, passive immunisation, and exploratory algorithm-classified guideline concordance in the overall cohort. Analyses of documented active vaccination by stated intention were restricted to the clinically indicated subgroup. To examine the intention–vaccination gap, an exploratory multivariable logistic regression was conducted among 333 participants who were both willing to vaccinate and clinically indicated for active vaccination. Documented vaccination within 7 days was the binary outcome. Model 1 adjusted for sociodemographic, clinical, healthcare setting, and previous immunisation history factors, and Model 2 additionally included the three BeSD constructs. Adjusted ORs with 95% CIs were reported. Given the 100 vaccination events, results were interpreted cautiously. Sensitivity analyses included site-level cluster-robust standard errors, common-method-bias checks, logistic regression, and SEM excluding the healthcare-provider recommendation item, a duplicate wound-category adjudication sample, and analysis by immunisation-history classification. Inter-rater agreement was summarised using exact percentage agreement and unweighted Cohen’s kappa for the four-category classification. Linear weighted kappa was additionally calculated among records for which both reviewers assigned a determinate wound category. Ninety-five percent confidence intervals were reported for both kappa estimates. Alternative-outcome models were exploratory because the temporal sequence between questionnaire completion, clinician recommendation, and documented clinical management could not be confirmed for all participants. Given the limited number of outcome events relative to covariate parameters, these models were also interpreted cautiously. Alternative-outcome estimates were reported only when obtained directly from fitted multivariable logistic regression models.

All analyses were performed using IBM SPSS Statistics 27.0, IBM SPSS AMOS 26.0, and R 4.4.1 with relevant packages for sensitivity analyses. A two-sided *p* value <0.05 was considered statistically significant.

## 3 Results

### 3.1 Participant characteristics, intention, and behaviour

Across the 12 participating sites, 742 patients were screened for eligibility, of whom 706 (95.1%) met the eligibility criteria. Among eligible patients, 18 (2.5%) declined participation, and 16 (2.3%) did not complete recruitment for other reasons. Of the latter, 11 left the care site after eligibility screening but before completion of study procedures, and five did not complete recruitment for other reasons. Overall, 672 participants completed the questionnaire and entered quality control, corresponding to a participation rate of 95.2% among eligible patients. Fifteen questionnaires (2.2% of completed questionnaires) were excluded because of invalid responses or incomplete key data. The final analytic cohort, therefore, comprised 657 participants, representing 97.8% of completed questionnaires and 93.1% of all eligible patients (Supplementary Figure S1; Supplementary Table S1).

A total of 657 valid questionnaires were included in the overall analytic cohort. Of these participants, 286 (43.5%) reported animal-related injuries and 371 (56.5%) reported non-animal-related injuries. The recruitment setting differed substantially between participants with animal-related and non-animal-related injuries [*χ*^2^(3) = 64.38, *p* < 0.001; Cramér’s V = 0.313]. Among participants with animal-related injuries, 120/286 (42.0%) were recruited from animal-injury clinics, compared with 55/371 (14.8%) of participants with non-animal-related injuries. Conversely, emergency departments accounted for 21.0% of animal-related injuries and 34.0% of non-animal-related injuries, while surgical outpatient clinics accounted for 17.5 and 29.6%, respectively. This distribution is detailed in Supplementary Table S1. Previous tetanus immunisation was formally verified in 190 participants (28.9%); 121 participants (18.4%) reported no previous vaccination or a most recent dose more than 10 years prior, and 346 (52.7%) had an unknown or uncertain immunisation history. Overall, 341 participants (51.9%) reported a willingness to receive a tetanus-containing vaccination. Among the 126 participants with documented active vaccination within 7 days, 121 (96.0%) received vaccination on the day of the trauma-care encounter, while 5 (4.0%) received vaccination during days 1–7 after the encounter. Among participants without documented vaccination within 7 days, no additional tetanus-containing vaccinations were identified through 30 June 2026 based on provincial immunisation records. Willingness was more frequent among participants with animal-related than non-animal-related injuries (61.5% vs. 44.5%; Table 2). Willingness groups also differed by residence, education, monthly income, recruitment setting, wound category, and previous tetanus immunisation history. Age, sex, and recruitment institution did not differ materially between groups (Table 2).

**Table 2:** Characteristics of participants according to vaccination intention in the overall analytic cohort (*n* = 657).

Characteristic	Category	Overall cohort (*n* = 657), *n* (%)	Willing participants (*n* = 341), *n* (%)	Not willing or uncertain participants (*n* = 316), *n* (%)	*p* value
Trauma type	Animal-related injury	286 (43.5%)	176 (51.6%)	110 (34.8%)	<0.001
	Non-animal-related injury	371 (56.5%)	165 (48.4%)	206 (65.2%)	
Sex	Male	370 (56.3%)	189 (55.4%)	181 (57.3%)	0.689
	Female	287 (43.7%)	152 (44.6%)	135 (42.7%)	
Age group, years	18–29	141 (21.5%)	73 (21.4%)	68 (21.5%)	0.792
	30–44	209 (31.8%)	114 (33.4%)	95 (30.1%)	
	45–59	183 (27.9%)	93 (27.3%)	90 (28.5%)	
	≥60	124 (18.9%)	61 (17.9%)	63 (19.9%)	
Residence	Urban	334 (50.8%)	159 (46.6%)	175 (55.4%)	0.030
	Rural	323 (49.2%)	182 (53.4%)	141 (44.6%)	
Education	Junior middle school or below	245 (37.3%)	108 (31.7%)	137 (43.4%)	0.002
	Senior high school or technical secondary school	213 (32.4%)	112 (32.8%)	101 (32.0%)	
	College or above	199 (30.3%)	121 (35.5%)	78 (24.7%)	
Monthly income, yuan	< 3,000	206 (31.4%)	96 (28.2%)	110 (34.8%)	0.008
	3,000–5,999	310 (47.2%)	156 (45.7%)	154 (48.7%)	
	≥6,000	141 (21.5%)	89 (26.1%)	52 (16.5%)	
Recruitment institution	County-level or above hospital	384 (58.4%)	202 (59.2%)	182 (57.6%)	0.869
	Community health service centre	142 (21.6%)	71 (20.8%)	71 (22.5%)	
	Township hospital	131 (19.9%)	68 (19.9%)	63 (19.9%)	
Recruitment sitting	Emergency department	186 (28.3%)	91 (26.7%)	95 (30.1%)	0.001
	Surgical outpatient clinic	160 (24.4%)	74 (21.7%)	86 (27.2%)	
	Animal-injury clinic	175 (26.6%)	113 (33.1%)	62 (19.6%)	
	Primary vaccination or wound-care clinic	136 (20.7%)	63 (18.5%)	73 (23.1%)	
Wound category	Clean minor wound	190 (28.9%)	93 (27.3%)	97 (30.7%)	0.010
	Contaminated or deep wound	328 (49.9%)	160 (46.9%)	168 (53.2%)	
	Severely contaminated or complex wound	139 (21.2%)	88 (25.8%)	51 (16.1%)	
Previous tetanus immunisation	Formally verified previous vaccination	190 (28.9%)	83 (24.3%)	107 (33.9%)	0.007
	Self-reported no vaccination or most recent dose >10 years previously	121 (18.4%)	59 (17.3%)	62 (19.6%)	
	Unknown or uncertain immunisation history	346 (52.7%)	199 (58.4%)	147 (46.5%)	

Percentages in the willingness columns are column percentages. Category-specific willingness rates use the category total as the denominator. For example, willingness was reported by 176/286 (61.5%) participants with animal-related injuries and 165/371 (44.5%) participants with non-animal-related injuries. p values were calculated using chi-square tests with continuity correction for 2×2 tables or Fisher’s exact tests, as appropriate. There were no missing data for the variables presented in Table 2. Unknown or uncertain immunisation history was treated as an analytic category rather than as missing data.

### 3.2 Measurement model validation

All 18 BeSD items loaded onto their prespecified latent constructs, with standardised factor loadings ranging from 0.64 to 0.87 (Table 1). Internal consistency was satisfactory across all four constructs. Cronbach’s α ranged from 0.846 to 0.883, and McDonald’s ω ranged from 0.848 to 0.883. Composite reliability ranged from 0.847 to 0.883, and average variance extracted ranged from 0.522 to 0.715, supporting convergent validity. Pairwise HTMT ratios ranged from 0.309 to 0.525, with a maximum value of 0.525, supporting discriminant validity (Table 3 and Supplementary Table S4).

**Table 3:** Reliability, convergent validity, discriminant validity, CFA fit, and sensitivity analysis of the social-processes construct (*n* = 657).

Panel A. Primary measurement model			
Domain	Indicator	Estimate	Reference benchmark
Internal consistency	Thinking and feeling, Cronbach’s α	0.866	≥0.70
	Thinking and feeling, McDonald’s ω	0.868	≥0.70
	Social processes, Cronbach’s α	0.846	≥0.70
	Social processes, McDonald’s ω	0.848	≥0.70
	Practical accessibility, Cronbach’s α	0.863	≥0.70
	Practical accessibility, McDonald’s ω	0.864	≥0.70
	Vaccination intention, Cronbach’s α	0.883	≥0.70
	Vaccination intention, McDonald’s ω	0.883	≥0.70
Convergent validity	Thinking and feeling, CR / AVE	0.867/0.522	CR ≥ 0.70; AVE ≥ 0.50
	Social processes, CR / AVE	0.847/0.582	CR ≥ 0.70; AVE ≥ 0.50
	Practical accessibility, CR / AVE	0.863/0.558	CR ≥ 0.70; AVE ≥ 0.50
	Vaccination intention, CR / AVE	0.883/0.715	CR ≥ 0.70; AVE ≥ 0.50
Discriminant validity	Maximum HTMT between constructs	0.525	<0.85
CFA model fit	*χ*^2^ (df)	309.73 (129)	—
	*p* value	<0.001	—
	*χ*^2^/df	2.401	<3.00
	CFI	0.949	≥0.90
	TLI	0.941	≥0.90
	RMSEA	0.046	<0.08
	RMSEA 90% CI	0.040–0.053	—
	SRMR	0.045	<0.08

Panel B. Sensitivity analysis of the social-processes construct excluding SP3		
Indicator	Primary model	Excluding SP3
SP1 standardised loading	0.78	0.79
SP2 standardised loading	0.74	0.75
SP3 standardised loading	0.82	—
SP4 standardised loading	0.71	0.72
Cronbach’s α	0.846	0.787
CR	0.847	0.798
AVE	0.582	0.568
*χ*^2^/df	2.401	2.360
CFI	0.949	0.951
TLI	0.941	0.943
RMSEA	0.046	0.045
SRMR	0.045	0.044

The primary CFA was conducted in the full analytic cohort (*n* = 657). In the sensitivity model, SP3 (healthcare-provider recommendation) was removed from the social-processes construct while the remaining measurement specification was retained. The full pairwise HTMT matrix is presented in Supplementary Table S4. α, Cronbach’s alpha; ω, McDonald’s omega; CR, composite reliability; AVE, average variance extracted; HTMT, heterotrait-to-monotrait ratio; CFA, confirmatory factor analysis; CFI, comparative fit index; TLI, Tucker–Lewis index; RMSEA, root mean square error of approximation; SRMR, standardised root mean square residual; CI, confidence interval.

The prespecified four-factor CFA showed acceptable overall fit: *χ*^2^(129) = 309.73, *p* < 0.001; *χ*^2^/df = 2.401; CFI = 0.949; TLI = 0.941; RMSEA = 0.046 (90% CI, 0.040–0.053); and SRMR = 0.045 (Table 3). Complete BeSD item data were available for all 657 participants (657/657, 100%), and no item-level imputation was required; therefore, the CFA and subsequent SEM used the full analytic sample (*n* = 657). Taken together, the item loadings, internal consistency estimates, convergent and discriminant validity indices, and overall CFA fit supported retention of the prespecified four-factor measurement structure.

Within the social-processes construct, healthcare-provider recommendation (SP3) showed a strong standardised factor loading of 0.82, compared with 0.78, 0.74, and 0.71 for SP1, SP2, and SP4, respectively. The four-item social-processes construct demonstrated good internal consistency and convergent validity (Cronbach’s α = 0.846, CR = 0.847, AVE = 0.582). In the sensitivity CFA excluding SP3, the loadings of the remaining indicators were 0.79, 0.75, and 0.72, while Cronbach’s α, CR, and AVE remained acceptable at 0.787, 0.798, and 0.568, respectively. Overall model fit changed only minimally after exclusion of SP3 (*χ*^2^/df = 2.360, CFI = 0.951, TLI = 0.943, RMSEA = 0.045, SRMR = 0.044), indicating that removal of SP3 did not materially alter the measurement structure.

### 3.3 Hierarchical logistic regression for binary vaccination intention

Model performance improved across the hierarchical logistic models (Nagelkerke R-squared: 0.050 in Model 1, 0.114 in Model 2, and 0.280 in Model 3; Table 4). In the fully adjusted model, animal-related injury (aOR = 1.69, 95% CI 1.17–2.43; *p* = 0.005), thinking and feeling (aOR = 1.46, 95% CI 1.18–1.80; *p* < 0.001), social processes (aOR = 1.68, 95% CI 1.36–2.07; *p* < 0.001), and practical accessibility (aOR = 2.08, 95% CI 1.66–2.62; *p* < 0.001) were positively associated with affirmative vaccination intention. Contaminated or deep wounds were inversely associated with affirmative intention compared with clean minor wounds (aOR = 0.63, 95% CI 0.41–0.96; *p* = 0.030). The maximum VIF was 1.76. In the sensitivity analysis accounting for clustering within the 12 healthcare sites, estimates remained materially consistent for animal-related injury (aOR 1.69, 95% CI 1.18–2.42), thinking and feeling (aOR 1.45, 95% CI 1.17–1.79), social processes (aOR 1.68, 95% CI 1.40–2.01), and practical accessibility (aOR 2.08, 95% CI 1.68–2.58), with no substantive change in the direction or interpretation of the primary findings (Supplementary Table S5).

**Table 4:** Hierarchical multivariable logistic regression for binary tetanus vaccination intention in the overall analytic cohort (*n* = 657).

Variable	Model 1 aOR (95% CI)	*p* value	Model 2 aOR (95% CI)	*p* value	Model 3 aOR (95% CI)	*p* value
Female vs. male	1.09 (0.80–1.50)	0.591	1.09 (0.79–1.52)	0.606	1.10 (0.77–1.57)	0.602
Age 30–44 vs. 18–29 years	1.17 (0.75–1.81)	0.485	1.25 (0.79–1.97)	0.338	1.19 (0.72–1.95)	0.497
Age 45–59 vs. 18–29 years	0.97 (0.62–1.52)	0.894	1.01 (0.63–1.61)	0.967	0.98 (0.59–1.61)	0.933
Age ≥60 vs. 18–29 years	0.88 (0.54–1.45)	0.612	0.94 (0.56–1.56)	0.813	0.72 (0.41–1.26)	0.256
Senior high school/technical secondary school vs. junior middle school or below	1.37 (0.94–1.99)	0.102	1.26 (0.85–1.86)	0.247	0.98 (0.64–1.50)	0.920
College or above vs. junior middle school or below	1.87 (1.27–2.75)	0.001	1.64 (1.09–2.47)	0.018	0.85 (0.53–1.35)	0.492
Monthly income 3,000–5,999 vs. <3,000 yuan	1.14 (0.80–1.63)	0.473	1.10 (0.76–1.60)	0.616	0.86 (0.57–1.30)	0.472
Monthly income ≥6,000 vs. <3,000 yuan	1.84 (1.17–2.88)	0.008	1.64 (1.03–2.62)	0.038	0.84 (0.50–1.42)	0.517
Urban vs. rural residence	0.79 (0.58–1.09)	0.143	0.83 (0.60–1.16)	0.268	1.41 (0.97–2.05)	0.073
Animal-related injury vs. non-animal-related injury	NA	—	1.77 (1.27–2.47)	<0.001	1.69 (1.17–2.43)	0.005
Contaminated/deep wound vs. clean minor wound	NA	—	0.82 (0.56–1.19)	0.302	0.63 (0.41–0.96)	0.030
Severe/complex wound vs. clean minor wound	NA	—	1.33 (0.83–2.15)	0.243	0.67 (0.39–1.14)	0.139
County-level or above hospital vs. primary institution	NA	—	1.03 (0.74–1.44)	0.862	0.93 (0.65–1.34)	0.711
Surgical outpatient clinic vs. emergency department	NA	—	0.98 (0.63–1.54)	0.929	1.47 (0.89–2.43)	0.128
Animal-injury clinic vs. emergency department	NA	—	1.60 (1.02–2.51)	0.041	1.21 (0.75–1.97)	0.437
Primary vaccination or wound-care clinic vs. emergency department	NA	—	0.83 (0.52–1.32)	0.433	1.04 (0.62–1.73)	0.883
Self-reported no vaccination or most recent dose >10 years previously vs. formally verified previous vaccination	NA	—	1.30 (0.80–2.11)	0.289	1.30 (0.77–2.19)	0.330
Unknown or uncertain immunisation history vs. formally verified previous vaccination	NA	—	1.44 (0.98–2.12)	0.064	0.98 (0.64–1.51)	0.942
Thinking and feeling score	NA	—	NA	—	1.46 (1.18–1.80)	<0.001
Social processes score	NA	—	NA	—	1.68 (1.36–2.07)	<0.001
Practical accessibility score	NA	—	NA	—	2.08 (1.66–2.62)	<0.001
Model performance: Nagelkerke *R*^2^	0.050	—	0.114	—	0.280	—
Multicollinearity check: Maximum VIF	1.72	—	1.74	—	1.76	—

Model 1 adjusted for sex, age group, education, monthly income, and residence. Model 2 additionally included injury type, wound category, recruitment institution, recruitment setting, and previous tetanus immunisation history. Model 3 further included thinking and feeling, social processes, and practical accessibility. Reference categories were male sex, age 18–29 years, junior middle school or below, monthly income <3,000 yuan, rural residence, non-animal-related injury, clean minor wound, primary institution, emergency department, and formally verified previous tetanus immunisation. For the BeSD construct scores, aORs represent the association per one-point increase in the corresponding 1–5 mean score. — indicates that the predictor was not included in the corresponding model. Analyses were conducted in the overall analytic cohort (*n* = 657).

The alternative-outcome sensitivity analysis using the mean three-item vaccination-intention score showed a materially consistent pattern. Thinking and feeling (B = 0.170, 95% CI 0.111–0.229), social processes (B = 0.240, 95% CI 0.181–0.299), and practical accessibility (B = 0.330, 95% CI 0.271–0.389) remained positively associated with stronger vaccination intention. Animal-related injury was also positively associated with the continuous intention score (B = 0.170, 95% CI 0.052–0.288), whereas contaminated or deep wounds remained inversely associated (B = −0.110, 95% CI − 0.208 to −0.012). Thus, the direction and substantive interpretation of the main associations were not materially altered by using the three-item intention score instead of the binary global outcome (Supplementary Table S6).

### 3.4 Structural equation model

The structural equation model showed an acceptable overall fit: *χ*^2^(129) = 309.73, *p* < 0.001; *χ*^2^/df = 2.401; CFI = 0.949; TLI = 0.941; RMSEA = 0.046 (90% CI, 0.040–0.053); and SRMR = 0.045. The model accounted for 56.4% of the variance in latent vaccination intention (Figure 1). Practical accessibility showed the largest modelled association with latent intention (standardised coefficient = 0.392, 95% CI 0.329–0.456), followed by social processes (0.268, 95% CI 0.201–0.336) and thinking and feeling (0.178, 95% CI 0.112–0.245). Thinking and feeling was associated with social processes and practical accessibility, and social processes was associated with practical accessibility (Table 5).

**Figure 1:**
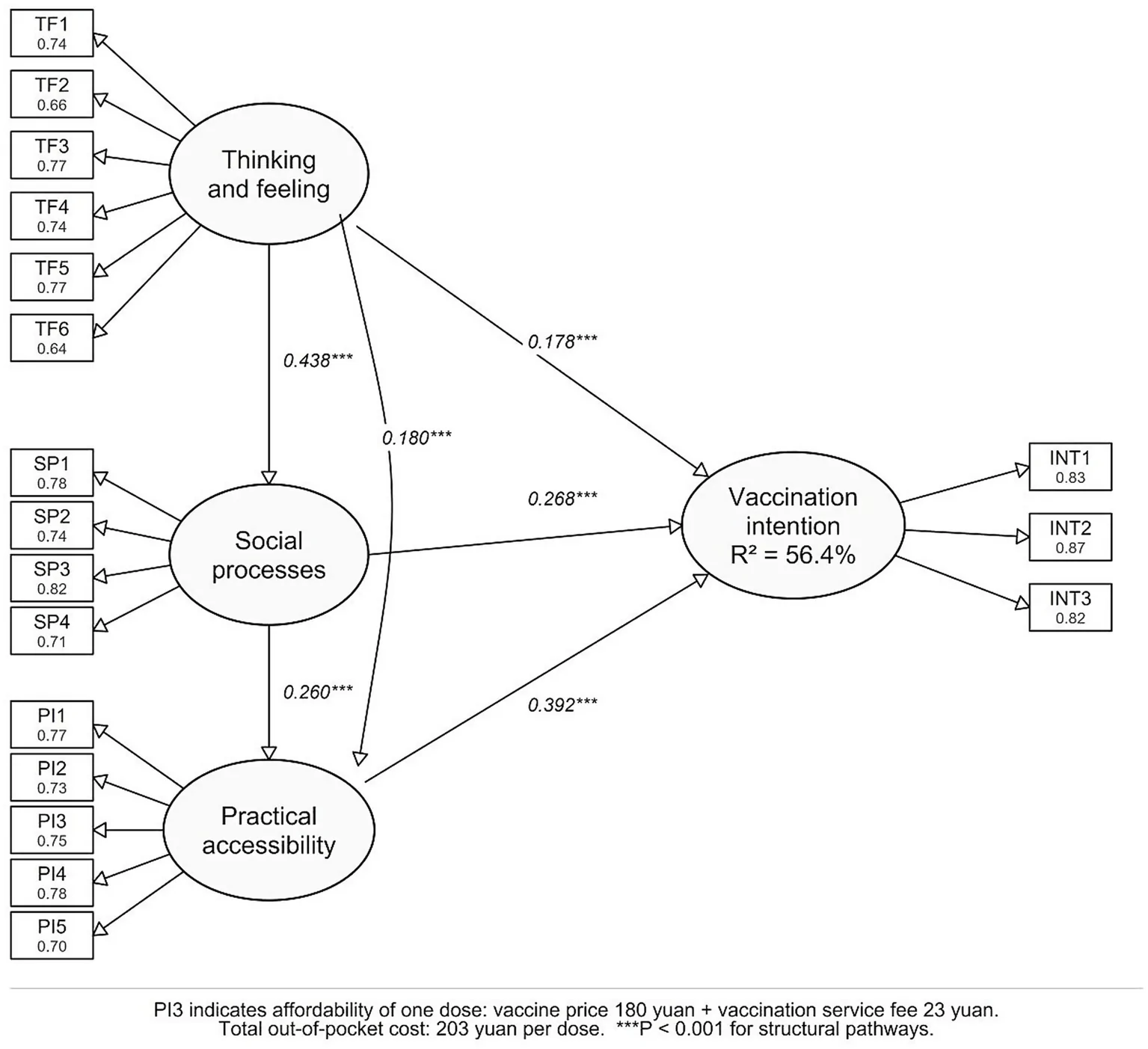
Cross-sectional structural equation model of BeSD constructs associated with tetanus vaccination intention among trauma-exposed adults. Rectangles represent observed questionnaire items, ellipses represent latent constructs, and arrows show standardised factor loadings or modelled structural coefficients. Practical accessibility was coded so that higher scores indicated fewer practical barriers and greater perceived accessibility, convenience, affordability, and service trust. PI3 indicates the affordability of one dose, defined as 180 yuan for the vaccine price plus 23 yuan for the vaccination service fee. Standardised coefficients are interpreted as theory-consistent associations rather than causal effects. **p < 0.01; ***p < 0.001.

**Table 5:** Standardised coefficients for prespecified associations in the structural equation model in the overall analytic cohort (*n* = 657).

Prespecified model relation	Standardised coefficient	95% CI	SE	*p* value
Thinking and feeling - > Social processes	0.438	0.369–0.507	0.035	<0.001
Social processes - > Practical accessibility	0.26	0.181–0.339	0.04	<0.001
Thinking and feeling - > Practical accessibility	0.18	0.101–0.259	0.04	<0.001
Thinking and feeling - > Vaccination intention	0.178	0.112–0.245	0.034	<0.001
Social processes - > Vaccination intention	0.268	0.201–0.336	0.034	<0.001
Practical accessibility - > Vaccination intention	0.392	0.329–0.456	0.032	<0.001

Common-method-bias checks did not indicate a dominant common-method factor. In sensitivity analyses excluding the healthcare-provider-recommendation item, the logistic association for social processes remained positive (OR 1.55, 95% CI 1.26–1.91; *p* < 0.001); estimates for thinking and feeling (OR 1.44, 95% CI 1.16–1.78), practical accessibility (OR 2.04, 95% CI 1.62–2.57), and animal-related injury (OR 1.67, 95% CI 1.15–2.41) were directionally consistent. The corresponding SEM also retained positive associations of social processes (standardised beta 0.24, 95% CI 0.17–0.31) and practical accessibility (0.38, 95% CI 0.31–0.45) with latent intention (all *p* < 0.001; Supplementary Table S5).

Documentation of vaccination status within 7 days was available for the overall analytic cohort. Among the 657 participants, 126 (19.2%) had documented tetanus-containing vaccination within 7 days. Of these 126 participants, 121 (96.0%) were vaccinated on day 0, and 5 (4.0%) were vaccinated on days 1–7, corresponding to 121/657 (18.4%) and 5/657 (0.8%) of the overall cohort, respectively. Among the remaining 531 participants without documented vaccination within 7 days, no subsequent tetanus-containing vaccination was identified in the provincial immunisation information system through 30 June 2026. In addition, 93 participants (14.2%) received passive immunisation, and 119/657 (18.1%) met the prespecified exploratory algorithm-classified guideline-concordance definition. Active vaccination was more frequent after animal-related injuries than after non-animal-related injuries (27.6% vs. 12.7%; crude OR 2.63, 95% CI 1.76–3.93; *p* < 0.001), whereas passive immunisation and algorithm-classified guideline concordance did not differ significantly (15.7% vs. 12.9%, *p* = 0.365; 19.2% vs. 17.3%, *p* = 0.581). The association was attenuated after adjustment (Model A: aOR 1.94, 95% CI 1.22–3.08, *p* = 0.005; Model B: aOR 1.72, 95% CI 1.06–2.79, *p* = 0.028). Full model estimates are presented in Supplementary Table S8. Fifteen participants without a guideline-based indication for active vaccination were retained in these overall analyses but excluded from the intention-by-vaccination comparison. These participants comprised nine with contaminated, deep, severely contaminated, or complex wounds and a completed primary series with the most recent dose within 5 years, and six with clean minor wounds and a completed primary series with the most recent dose within 10 years. Among the 642 clinically indicated participants, 333 expressed willingness; 100/333 (30.0%) had documented active vaccination within 7 days, and 233/333 (70.0%) did not. This latter proportion describes documented vaccination status among participants with a guideline-based indication and should not be interpreted as a general measure of service failure (Table 6).

**Table 6:** Documented post-exposure interventions and vaccination status by stated intention.

Panel A. Outcomes in the overall analytic cohort (*n* = 657)				
Behavioural outcome	Overall	Animal-related injury	Non-animal-related injury	*p* value
Documented tetanus-containing vaccination within 7 days	126 (19.2%)	79/286 (27.6%)	47/371 (12.7%)	<0.001
Passive immunisation product received	93 (14.2%)	45/286 (15.7%)	48/371 (12.9%)	0.365
Algorithm-classified guideline concordance	119 (18.1%)	55/286 (19.2%)	64/371 (17.3%)	0.581

Panel B. Documented active vaccination by stated intention in participants with a guideline-based indication for active tetanus vaccination (*n* = 642)				
Intention–vaccination outcome	Overall	Animal-related injury	Non-animal-related injury	*p* value
Willing but not documented as vaccinated within 7 days	233/333 (70.0%)	103/171 (60.2%)	130/162 (80.2%)	<0.001
Willing and documented as vaccinated within 7 days	100/333 (30.0%)	68/171 (39.8%)	32/162 (19.8%)	<0.001
Not willing/uncertain but documented as vaccinated within 7 days	26/309 (8.4%)	11/108 (10.2%)	15/201 (7.5%)	0.544

Panel C. Sensitivity analysis of algorithm-classified guideline concordance by immunisation-history verification		
Analysis population	Algorithm-classified guideline concordance, n/N	% (95% CI)
Overall analytic cohort	119/657	18.1 (15.36–21.24)
Formally verified previous immunisation history	45/190	23.7 (18.20–30.21)

Panel A uses the overall analytic cohort (*n* = 657). Vaccination timing was summarised among participants with documented active vaccination within 7 days after trauma exposure. Day 0 vaccination refers to vaccination administered on the day of the index trauma-care encounter, and days 1–7 refer to vaccination documented within the subsequent 7 days.

Among the 333 willing participants with an indication for active vaccination, greater practical accessibility (aOR 1.62, 95% CI 1.22–2.15, *p* < 0.001) and animal-related injury (aOR 1.84, 95% CI 1.02–3.31, *p* = 0.042) were independently associated with documented vaccination within 7 days. Social processes showed a positive but non-significant association (aOR 1.29, 95% CI 0.96–1.73, *p* = 0.090), while thinking and feeling were not associated (aOR 1.10, 95% CI 0.80–1.51, *p* = 0.551). Other measured sociodemographic, wound, healthcare-setting, and immunisation-history factors were not independently associated. Full estimates are provided in Supplementary Table S9.

Sensitivity analyses supported cautious interpretation of the algorithm-classified clinical outcome. In the primary immunisation-history sensitivity analysis, which was restricted to 190 participants with formally verified previous immunisation information, 45 out of 190 participants met the algorithm-classified guideline-concordance definition (23.7, 95% CI 18.20–30.21), compared with 119 out of 657 (18.1, 95% CI 15.36–21.24) in the overall cohort. In an additional descriptive analysis, 64 out of 311 participants (20.6, 95% CI 16.46–25.42) with a non-unknown questionnaire-based immunisation-history classification met the algorithm-classified guideline-concordance definition. This analysis of 311 participants included those with formally verified previous vaccination and those in the self-reported inadequate-history category; it was not a fully verified immunisation-history analysis. The corresponding proportions were 19 out of 121 (15.7, 95% CI 10.29–23.23) in the self-reported inadequate-history category and 55 out of 346 (15.9, 95% CI 12.42–20.12) among those with an unknown or uncertain history (Supplementary Table S7). In the stratified duplicate-review sample of 197 source records, the four-category wound classification had 86.8% agreement and a Cohen’s kappa of 0.80 (95% CI 0.72–0.87). Among 184 records with determinate categories assigned by both reviewers, the linear weighted kappa was 0.84 (95% CI 0.77–0.91). Twenty-six records required adjudication by a third reviewer; 10 remained indeterminate. In the 187 determinate adjudicated records, 39 (20.9, 95% CI 15.7–27.3) met the algorithm-classified guideline-concordance definition.

## 4 Discussion

This study highlights that tetanus vaccination decisions after trauma exposure are shaped by multiple behavioural and healthcare-context factors rather than by a single factor. The findings suggest that perceptions of service accessibility, social influences, and individual beliefs may jointly contribute to how patients approach vaccination decisions during trauma care. Differences observed across trauma contexts should be interpreted within the broader healthcare pathway, as injury type itself may reflect differences in clinical settings, provider practices, and prevention opportunities. These findings should be interpreted alongside clinical indication, as documented vaccination is not expected for every trauma-exposed adult. The gap between willingness and documented vaccination should therefore be understood in the context of clinical indication and healthcare delivery processes. Within this clinically indicated subgroup, the absence of documented vaccination within 7 days should be viewed as a descriptive finding of early documented vaccine uptake rather than direct evidence of failed vaccine delivery. Most documented vaccinations occurred during the index trauma-care encounter: 121/126 (96.0%) were administered on day 0, whereas only 5/126 (4.0%) occurred on days 1–7. Furthermore, no subsequent tetanus-containing vaccination was identified among the 531 participants without documented vaccination within 7 days when provincial immunisation records were searched through 30 June 2026. These findings support the use of the 7-day interval as an operational measure of early documented vaccination in this cohort, although the duration of extended record ascertainment varied according to recruitment date.

The BeSD findings highlight an important conceptual distinction between individual behavioural or attitudinal barriers and healthcare-system or service-access barriers. Perceived practical accessibility was the construct most strongly associated with affirmative vaccination intention. This construct included service availability, time convenience, the local out-of-pocket cost of 203 yuan per dose, process convenience, and trust in service delivery. Thus, lower willingness or documented vaccination uptake in trauma-care settings should not automatically be interpreted as vaccine refusal or hesitancy alone, as documented vaccination may also be associated with practical constraints and accessibility within the care environment (7, 24–26). Practical constraints related to cost, service availability, workflow, and opportunities for same-visit vaccination may influence whether vaccination is perceived as feasible, while uncertainty in previous immunisation records may further complicate clinical decision-making. These responses may have reflected perceptions formed before, during, or after components of the clinical encounter; therefore, they should be interpreted as service-context correlates rather than causal healthcare-system effects of vaccination. The robustness of these associations to outcome operationalisation was supported by the sensitivity analysis using the three-item continuous intention score, which reproduced the direction and overall pattern observed with the primary binary outcome.

Descriptive analyses showed differences by residence, education, monthly income, and wound category. These associations weakened after adjustment for trauma characteristics, healthcare factors, and BeSD constructs. This pattern does not render demographic factors irrelevant. Instead, these demographic differences may coexist with, or be statistically attenuated after accounting for, more proximal service-context perceptions. Evidence from vaccine-hesitancy interventions similarly indicates that information alone has limited value when practical barriers remain (27).

Provider recommendation should be interpreted as a contextual feature of the care episode rather than as a stand-alone behavioural factor. In this setting, provider recommendation is closely linked to patient perceptions and clinical decision-making occurring during the same encounter. Prior work has reported associations between provider confidence, communication, and recommendation practices and vaccine hesitancy (28), while meta-analytic evidence has also linked provider communication with vaccine uptake, although much of this evidence comes from other vaccines and populations (29). Healthcare provider recommendation was retained within the social processes construct because the WHO BeSD framework conceptualises health worker recommendation as a social influence on vaccination behaviour (14). However, in trauma-care settings, this item may also reflect aspects of healthcare delivery and patient-provider interaction. Therefore, healthcare provider recommendation should be interpreted as a context-dependent factor that integrates both social influence and clinical communication. Nevertheless, because provider recommendation occurred within the trauma-care encounter, it may also partly capture clinical service delivery. Importantly, the associations for social processes remained directionally consistent after SP3 was removed in both logistic regression (OR 1.55, 95% CI 1.26–1.91) and SEM (standardised *β* = 0.24, 95% CI 0.17–0.31), supporting the robustness of the main findings while retaining appropriate caution regarding construct overlap and temporal ordering.

The SEM model should not be read as a timeline. The observed coefficients describe cross-sectional associations among thinking and feeling, social processes, practical accessibility, and latent vaccination intention, and do not establish temporal direction or causation. These findings are therefore best interpreted as a cross-sectional pattern of cognitive, social, and service-context correlates rather than as a sequence of behavioural mechanisms. Accordingly, any intervention implications should be considered hypothesis-generating rather than as evidence that modifying one construct would necessarily change vaccination intention. This interpretation is consistent with vaccination frameworks that consider confidence, complacency, constraints, calculation, and access within the same behavioural context (24, 25).

The behavioural outcomes demonstrated a substantial discrepancy between stated willingness and documented vaccination. A substantial gap remained between stated willingness and documented vaccination among clinically indicated participants. These findings suggest that factors contributing to vaccination intention may not fully overlap with those facilitating actual vaccine receipt. Transitioning from willingness to documented vaccination may depend more strongly on practical implementation factors, such as service accessibility, workflow coordination, and timely delivery of vaccination opportunities. These findings suggest that factors associated with forming vaccination intention may differ from those associated with translating intention into documented vaccination, with practical accessibility appearing particularly relevant to this transition (30). Several explanations remain plausible, including cost, time, service barriers, incomplete or delayed record capture, and differences between the study algorithm and a clinician’s assessment when relevant wound or immunisation history details were incompletely documented. However, given the cross-sectional care episode measures and limited number of vaccination events, these findings should be interpreted as exploratory associations rather than causal effects.

Practical accessibility included service availability, time and process convenience, affordability, and trust in service delivery. Its association with documented vaccination suggests that willingness alone may be insufficient when practical barriers remain (31). The association with animal-related injury may partly reflect differences in rabies post-exposure prophylaxis (PEP) care pathways rather than the injury type itself (11). These differences may include variation in healthcare settings, clinician recommendations, risk assessment processes, and availability of vaccination services. These findings remain exploratory and non-causal because temporal ordering could not be established, and only 100 vaccination events occurred.

Exploratory algorithm-classified guideline concordance was limited and should be interpreted cautiously because it depended on available wound documentation and immunisation history. This estimate reflects concordance between the prespecified study algorithm and documented post-exposure management based on the wound and immunisation history information available to the study. It should not be interpreted as indicating that only 18.1% of participants actually received appropriate prophylaxis or as a direct measure of clinician adherence to prophylaxis guidelines or service quality. Previous immunisation history was unknown or uncertain for 346 participants (52.7%), which directly limited the certainty of the algorithm-based classification. Sensitivity analyses further indicated that uncertainty in immunisation history and wound documentation remained important considerations when interpreting algorithm-classified concordance. Although additional verification procedures improved confidence in classification, residual uncertainty could not be completely eliminated because some clinical information was unavailable or incomplete. These findings support a cautious interpretation. They are compatible with variation in prophylaxis delivery, but they cannot fully distinguish this from residual uncertainty in wound assessment, immunisation history, or the clinical information recorded at the time of care. The higher documented active vaccination rate among participants with animal-related injuries should be interpreted cautiously. The crude association (OR 2.63, 95% CI 1.76–3.93) was attenuated after adjustment for sociodemographic, clinical, and healthcare-setting characteristics (aOR 1.94, 95% CI 1.22–3.08), and further after incorporating BeSD care-episode factors (aOR 1.72, 95% CI 1.06–2.79), suggesting that differences in case mix and care context may partly explain the observed contrast. Provider recommendation and service accessibility are recognised influences on vaccine uptake, while animal-related injuries may also enter distinct rabies post-exposure care pathways (32). These differences may include variation in healthcare settings, clinician recommendations, risk assessment processes, and availability of vaccination services. A residual association remained, but unmeasured differences in clinical workflow, clinician practice, or prophylaxis availability cannot be excluded. Moreover, passive immunisation and algorithm-classified guideline concordance did not differ significantly by injury type, indicating that higher active vaccine receipt did not necessarily represent more complete post-exposure prophylaxis.

These findings suggest that public health responses to post-traumatic tetanus prevention should address both behavioural and healthcare system barriers. At the behavioural level, patient education should explain not only the severity of tetanus but also why previous immunisation history changes post-exposure management. Clinics should standardise recommendations in emergency departments, surgical outpatient clinics, and primary wound-care settings, where vaccination decisions often occur during brief clinical encounters (28, 29). System changes may matter even more. Record linkage, stable vaccine supply, clear fee information, and same-visit vaccination pathways represent potential service-level targets for narrowing the gap between willingness and timely prophylaxis (26). Reminder and recall tools may also support follow-up when an indicated vaccination cannot be delivered during the initial trauma-care encounter (33).

Strengths included the application of the BeSD framework in trauma care, linkage of questionnaire data with documented clinical outcomes, and prespecified sensitivity analyses addressing wound classification and immunisation history uncertainty.

The study has several limitations. First, its cross-sectional design and the lack of systematic timestamps for questionnaire completion and clinical interactions prevent the establishment of temporal precedence and causal inference. Although questionnaires were intended to be administered early in the care episode, the sequence between questionnaire completion, physician assessment, provider recommendation, and vaccination administration could not be confirmed for all participants. Therefore, BeSD constructs, healthcare provider recommendation, and perceived accessibility should be interpreted as care episode correlates rather than confirmed antecedent exposures. The sensitivity analysis excluding healthcare provider recommendation reduced concern regarding this individual item but did not resolve the broader temporal ambiguity.

Second, participants recruited from the same healthcare site may have shared clinical workflows, provider practices, vaccine availability, and local healthcare characteristics, resulting in potential intra-site correlation. Although healthcare site cluster-robust sensitivity analyses produced materially similar estimates, only 12 sites were available, and residual site-level heterogeneity cannot be excluded.

Third, although duplicate review of a stratified sample of source records improved the reproducibility of wound classification, it did not replace the primary classification for the full cohort. In addition, previous immunisation history was unknown or uncertain for 346 participants, limiting the reliability of the algorithm-classified guideline concordance outcome. Therefore, the 18.1% concordance estimate should be interpreted as an exploratory classification based on available information rather than a direct measure of clinician adherence to prophylaxis guidelines.

Fourth, although vaccination status was verified through clinical records and the provincial immunisation information system, delayed registration or vaccination occurring outside linked records could have resulted in the misclassification of some participants as not vaccinated. The extended record search through 30 June 2026 provided supportive descriptive information but did not represent a uniform follow-up period.

Fifth, selection bias cannot be completely excluded despite consecutive recruitment and the low refusal rate. Participants from participating healthcare facilities or those willing to complete questionnaires may differ from other trauma-exposed individuals. In addition, recruitment settings differed by injury type, and residual confounding related to clinical workflow, provider practice, and availability of prophylaxis services may remain despite multivariable adjustment.

Finally, vaccination intention was self-reported and may have been affected by social-desirability bias, particularly because questionnaires were completed during healthcare encounters. Participants may have reported a greater willingness to vaccinate to align with perceived expectations from healthcare providers or investigators. Moreover, although the BeSD questionnaire showed satisfactory psychometric performance, it was developed and evaluated within a single regional trauma-exposed population, and further validation in independent populations is needed.

Furthermore, this study was conducted in a single city in Zhejiang Province, China, and included only adults seeking trauma-related healthcare. Therefore, the generalisability of our findings should be approached with caution. Healthcare access, out-of-pocket vaccination costs, clinical workflows, and immunisation-record systems vary substantially across different regions of China and internationally. In particular, the observed association between practical accessibility and vaccination intention may reflect the local healthcare delivery context, vaccination service organisation, and record-linkage infrastructure in Quzhou. Future multicentre studies involving diverse geographic regions and healthcare systems are needed to determine whether these findings are applicable beyond the current setting.

## 5 Conclusion

Tetanus vaccination intention after trauma exposure was associated with thinking and feeling, social processes, and perceived service accessibility during the care episode, with perceived service accessibility showing the largest association. These associations describe behavioural and service-context correlates within the trauma-care episode and should not be interpreted as establishing temporal ordering or causal relationships. Among participants already willing to vaccinate, greater practical accessibility was also associated with documented vaccination, suggesting that factors associated with vaccine uptake may differ from those associated with intention. Accordingly, low documented vaccination uptake should not be interpreted as vaccine hesitancy or refusal alone, but should be considered alongside practical and healthcare-system constraints. Documented active vaccination within 7 days was more frequent among participants with animal-related injuries, although the magnitude of this association was attenuated after adjustment for measured patient, clinical, healthcare-setting, and behavioural/service-context characteristics. This residual association should not be interpreted as evidence of a causal effect of injury type itself. The interpretation of documented vaccination and algorithm-classified guideline-concordance outcomes requires caution because these assessments depended on the completeness and reliability of routine wound documentation and previous immunisation history verification. Evaluation of post-exposure care should therefore integrate behavioural measures with verified immunisation history, wound assessment, and clinical indication.

## Data Availability

The datasets presented in this article are not readily available because they contain individual-level questionnaire, clinical, and immunisation information and are subject to institutional data-sharing restrictions. De-identified data supporting the findings may be made available upon reasonable request and subject to institutional approval. Requests to access the datasets should be directed to Zhiying Yin at yzy1815@sohu.com.
