# Supplementary material for: Behavioural and service-context correlates of tetanus vaccination intention after trauma exposure in China: a cross-sectional BeSD study
Source: Front Public Health. 2026 Sep 21;14:1941921. doi: 10.3389/fpubh.2026.1941921 (PMC13635121; doi:10.3389/fpubh.2026.1941921)
Supplement: Supplementary file 1 [file Data_Sheet_1.docx]

**Supplementary Figure S1. Flow of participants through the study and analytic cohorts.**

Note: Of 742 participants screened, 706 were eligible and 672 completed the questionnaire; 18 declined participation and 16 had other non-participation. Fifteen questionnaires were excluded during quality control, yielding an overall analytic cohort of 657. Wound category was verified against source medical records and previous tetanus immunisation history against available formal records. Histories lacking formal verification were classified as self-reported inadequate or unknown/uncertain and treated as lacking verified protection in the prophylaxis algorithm. Fifteen participants had no guideline-based indication for active tetanus vaccination after verification and were retained in the overall analytic cohort but excluded from the intention–vaccination gap denominator. The remaining 642 participants formed the clinically indicated subgroup.
