# Supplementary material for: Behavioural and service-context correlates of tetanus vaccination intention after trauma exposure in China: a cross-sectional BeSD study
Source: Front Public Health. 2026 Sep 21;14:1941921. doi: 10.3389/fpubh.2026.1941921 (PMC13635121; doi:10.3389/fpubh.2026.1941921)
Supplement: Supplementary file 2 [file Table_1.docx]

**Supplementary Table S1. Site-specific participant recruitment, analytic cohort derivation, and trauma-type distribution**

**Panel A. Site-specific screening, recruitment, and final analytic sample**

| **Site** | **Institution type** | **Recruitment setting** | **Screened, n** | **Eligible, n** | **Declined participation, n (%)¹** | **Other non-participation, n (%)¹** | **Completed questionnaire, n (%)¹** | **QC exclusions, n (%)²** | **Final analytic cohort, n (%)¹** | **Animal-related injury, n (%)³** | **Non-animal-related injury, n (%)³** |
| --- | --- | --- | --- | --- | --- | --- | --- | --- | --- | --- | --- |
| S1 | County-level or above hospital | Emergency department | 80 | 76 | 2 (2.6) | 2 (2.6) | 72 (94.7) | 2 (2.8) | 70 (92.1) | 22 (31.4) | 48 (68.6) |
| S2 | County-level or above hospital | Emergency department | 67 | 64 | 2 (3.1) | 1 (1.6) | 61 (95.3) | 1 (1.6) | 60 (93.8) | 19 (31.7) | 41 (68.3) |
| S3 | County-level or above hospital | Surgical outpatient clinic | 78 | 74 | 2 (2.7) | 2 (2.7) | 70 (94.6) | 2 (2.9) | 68 (91.9) | 22 (32.4) | 46 (67.6) |
| S4 | County-level or above hospital | Surgical outpatient clinic | 64 | 61 | 1 (1.6) | 1 (1.6) | 59 (96.7) | 1 (1.7) | 58 (95.1) | 18 (31.0) | 40 (69.0) |
| S5 | County-level or above hospital | Animal-injury clinic | 80 | 76 | 2 (2.6) | 2 (2.6) | 72 (94.7) | 2 (2.8) | 70 (92.1) | 49 (70.0) | 21 (30.0) |
| S6 | County-level or above hospital | Primary vaccination or wound-care clinic | 64 | 61 | 1 (1.6) | 1 (1.6) | 59 (96.7) | 1 (1.7) | 58 (95.1) | 25 (43.1) | 33 (56.9) |
| S7 | Community health service centre | Animal-injury clinic | 72 | 69 | 2 (2.9) | 2 (2.9) | 65 (94.2) | 1 (1.5) | 64 (92.8) | 43 (67.2) | 21 (32.8) |
| S8 | Community health service centre | Primary vaccination or wound-care clinic | 44 | 42 | 1 (2.4) | 1 (2.4) | 40 (95.2) | 1 (2.5) | 39 (92.9) | 16 (41.0) | 23 (59.0) |
| S9 | Community health service centre | Primary vaccination or wound-care clinic | 44 | 42 | 1 (2.4) | 1 (2.4) | 40 (95.2) | 1 (2.5) | 39 (92.9) | 15 (38.5) | 24 (61.5) |
| S10 | Township hospital | Emergency department | 63 | 60 | 1 (1.7) | 2 (3.3) | 57 (95.0) | 1 (1.8) | 56 (93.3) | 19 (33.9) | 37 (66.1) |
| S11 | Township hospital | Surgical outpatient clinic | 38 | 36 | 1 (2.8) | 0 (0.0) | 35 (97.2) | 1 (2.9) | 34 (94.4) | 10 (29.4) | 24 (70.6) |
| S12 | Township hospital | Animal-injury clinic | 48 | 45 | 2 (4.4) | 1 (2.2) | 42 (93.3) | 1 (2.4) | 41 (91.1) | 28 (68.3) | 13 (31.7) |
| **Total** | — | — | **742** | **706** | **18 (2.5)** | **16 (2.3)** | **672 (95.2)** | **15 (2.2)** | **657 (93.1)** | **286 (43.5)** | **371 (56.5)** |

**Panel B. Distribution of the final analytic cohort by trauma type and recruitment context**

| **Recruitment characteristic** | **Animal-related injury (n=286), n (%)** | **Non-animal-related injury (n=371), n (%)** | **χ² (df)** | **P value** | **Cramér's V** |
| --- | --- | --- | --- | --- | --- |
| Institution type |  |  | 5.82 (2) | 0.054 | 0.094 |
| County-level or above hospital | 155 (54.2) | 229 (61.7) |  |  |  |
| Community health service centre | 74 (25.9) | 68 (18.3) |  |  |  |
| Township hospital | 57 (19.9) | 74 (19.9) |  |  |  |
| Recruitment setting |  |  | 64.38 (3) | <0.001 | 0.313 |
| Emergency department | 60 (21.0) | 126 (34.0) |  |  |  |
| Surgical outpatient clinic | 50 (17.5) | 110 (29.6) |  |  |  |
| Animal-injury clinic | 120 (42.0) | 55 (14.8) |  |  |  |
| Primary vaccination or wound-care clinic | 56 (19.6) | 80 (21.6) |  |  |  |

Notes:

¹ Percentages for declined participation, other non-participation, completed questionnaire, and final analytic cohort were calculated using the number of eligible patients at the corresponding site as the denominator.

² Percentages for quality-control (QC) exclusions were calculated using the number of participants who completed the questionnaire as the denominator.

³ Percentages for animal-related and non-animal-related injuries were calculated using the final analytic sample at the corresponding site as the denominator.

“Other non-participation” comprised eligible individuals who did not complete recruitment for reasons other than explicit refusal; overall, this included 11 individuals who left the care site after eligibility screening but before completion of study procedures and five who did not complete recruitment for other reasons. QC exclusions comprised questionnaires that were invalid or contained incomplete key data. Differences in the distribution of the final analytic cohort by trauma type were assessed using chi-square tests, with Cramér's V reported as an effect-size measure.
