# Supplementary material for: Behavioural and service-context correlates of tetanus vaccination intention after trauma exposure in China: a cross-sectional BeSD study
Source: Front Public Health. 2026 Sep 21;14:1941921. doi: 10.3389/fpubh.2026.1941921 (PMC13635121; doi:10.3389/fpubh.2026.1941921)
Supplement: Supplementary file 3 [file Table_2.docx]

**Supplementary Table S2. Operational criteria for wound-category classification**

| Wound category | Operational definition |
| --- | --- |
| Clean minor wound | A superficial wound explicitly documented as clean and minor and limited to the epidermis or dermis. The source record had to provide sufficient information to exclude puncture or penetration, bite or saliva exposure, soil, faecal or foreign-material contamination, devitalised tissue, crush injury, open fracture, burn, frostbite, or other complex features. Records lacking sufficient documentation of these features were classified as indeterminate. |
| Contaminated or deep wound | Presence of at least one of the following without severe or complex features: puncture or penetrating injury; animal or human bite; saliva, soil, dust, faecal or foreign-material contamination; extension beyond the dermis; or explicit documentation of a contaminated or deep wound. |
| Severely contaminated or complex wound | Presence of devitalised or necrotic tissue, extensive crush injury, open fracture, substantial embedded contamination, retained foreign material, extensive burn or frostbite, clinically infected wound, or a wound requiring extensive debridement or surgical exploration. |
| Indeterminate | The medical record lacked sufficient information to determine wound depth, contamination, puncture or bite status, tissue viability or other defining characteristics, or contained conflicting information that could not be resolved. |

Note: Prespecified features were abstracted from source records. Severe or complex features took priority over contaminated or deep features. A wound was classified as clean and minor only when the source record contained sufficient information to exclude contaminated, deep, severe, or complex features.
