# Supplementary material for: Behavioural and service-context correlates of tetanus vaccination intention after trauma exposure in China: a cross-sectional BeSD study
Source: Front Public Health. 2026 Sep 21;14:1941921. doi: 10.3389/fpubh.2026.1941921 (PMC13635121; doi:10.3389/fpubh.2026.1941921)
Supplement: Supplementary file 4 [file Table_3.docx]

**Supplementary Table S3. Algorithm used to define algorithm-classified guideline-concordant tetanus post-exposure prophylaxis.**

| **Wound group** | **Clinical immunisation status for prophylaxis algorithm** | **Detailed verification rule** | **Active immunisation required** | **Passive immunisation required** | **Definition of algorithm-classified guideline-concordant prophylaxis** | |
| --- | --- | --- | --- | --- | --- | --- |
| Clean minor wound | Completed primary series + last dose within 10 years | Completed primary series verified; most recent tetanus-containing dose within 10 years | No | No | Concordant if neither active nor passive immunisation was documented. |  |
| Clean minor wound | Completed primary series + last dose >10 years | Completed primary series verified; most recent dose more than 10 years previously | Yes | No | Concordant if active immunisation was documented and passive immunisation was not documented. |  |
| Clean minor wound | Documented incomplete primary series or documented absence of previous tetanus vaccination | Formal records documented fewer than three tetanus-containing vaccine doses or documented no previous tetanus-containing vaccination. | Yes | No | Concordant if active immunisation was documented and passive immunisation was not documented. |  |
| Clean minor wound | Unknown or unverifiable history | No verifiable record of primary-series completion or most recent dose | Yes | No | Concordant if active immunisation was documented and passive immunisation was not documented. |  |
| Contaminated, deep, severely contaminated, or complex wound | Completed primary series + last dose within 5 years | Completed primary series verified; most recent tetanus-containing dose within 5 years | No | No | Concordant if neither active nor passive immunisation was documented. |  |
| Contaminated, deep, severely contaminated, or complex wound | Completed primary series + last dose >5 years | Completed primary series verified; most recent dose more than 5 years previously | Yes | No | Concordant if active immunisation was documented and passive immunisation was not documented. |  |
| Contaminated, deep, severely contaminated, or complex wound | Documented incomplete primary series or documented absence of previous tetanus vaccination | Formal records documented fewer than three tetanus-containing vaccine doses or documented no previous tetanus-containing vaccination. | Yes | Yes | Concordant only if both active and passive immunisation were documented. |  |
| Contaminated, deep, severely contaminated, or complex wound | Unknown or unverifiable history | No verifiable record of primary-series completion or most recent dose | Yes | Yes | Concordant only if both active and passive immunisation were documented. |  |

Note: The algorithm uses clinical information required for prophylaxis adjudication, rather than descriptive immunisation-history categories alone. Completed primary series was defined as documented receipt of at least three tetanus-containing vaccine doses, or verified completion of the age-appropriate childhood immunisation schedule. Self-reported inadequate histories without supporting formal records were assigned to the unknown or unverifiable category for the prophylaxis algorithm. Active immunisation referred to documented receipt of a tetanus-containing vaccine. Passive immunisation referred to documented receipt of tetanus immunoglobulin or tetanus antitoxin when required by the predefined algorithm.
