# Supplementary material for: Behavioural and service-context correlates of tetanus vaccination intention after trauma exposure in China: a cross-sectional BeSD study
Source: Front Public Health. 2026 Sep 21;14:1941921. doi: 10.3389/fpubh.2026.1941921 (PMC13635121; doi:10.3389/fpubh.2026.1941921)
Supplement: Supplementary file 5 [file Table_4.docx]

**Supplementary Table S4. Heterotrait–monotrait ratios among the four BeSD latent constructs**

| **Construct** | **Thinking and feeling** | **Social processes** | **Practical accessibility** | **Vaccination intention** |
| --- | --- | --- | --- | --- |
| Thinking and feeling | — |  |  |  |
| Social processes | 0.447 | — |  |  |
| Practical accessibility | 0.309 | 0.352 | — |  |
| Vaccination intention | 0.414 | 0.481 | 0.525 | — |

Note: Values represent heterotrait–monotrait ratios (HTMT) between latent constructs. All pairwise HTMT values were below the prespecified reference threshold of 0.85, supporting discriminant validity. TF, thinking and feeling; SP, social processes; PI, practical accessibility; INT, vaccination intention.
