# Supplementary material for: Behavioural and service-context correlates of tetanus vaccination intention after trauma exposure in China: a cross-sectional BeSD study
Source: Front Public Health. 2026 Sep 21;14:1941921. doi: 10.3389/fpubh.2026.1941921 (PMC13635121; doi:10.3389/fpubh.2026.1941921)
Supplement: Supplementary file 6 [file Table_5.docx]

**Supplementary Table S5. Sensitivity and alternative-outcome analyses in the overall analytic cohort (n=657)**

**Panel A1. Sensitivity analyses of the binary vaccination-intention model (n=657)**

| **Analysis** | **Predictor / structural path** | **Effect measure** | **Estimate** | **95% CI** | **P value** |
| --- | --- | --- | --- | --- | --- |
| Site-clustered robust standard errors | Animal-related injury | aOR | 1.69 | 1.18–2.42 | 0.004 |
| Site-clustered robust standard errors | Thinking and feeling | aOR | 1.45 | 1.17–1.79 | 0.001 |
| Site-clustered robust standard errors | Social processes | aOR | 1.68 | 1.40–2.01 | <0.001 |
| Site-clustered robust standard errors | Practical accessibility | aOR | 2.08 | 1.68–2.58 | <0.001 |
| Logistic model excluding healthcare-provider recommendation (SP3) | Animal-related injury | aOR | 1.67 | 1.15–2.41 | 0.007 |
| Logistic model excluding healthcare-provider recommendation (SP3) | Thinking and feeling | aOR | 1.44 | 1.16–1.78 | 0.001 |
| Logistic model excluding healthcare-provider recommendation (SP3) | Social processes excluding SP3 | aOR | 1.55 | 1.26–1.91 | <0.001 |
| Logistic model excluding healthcare-provider recommendation (SP3) | Practical accessibility | aOR | 2.04 | 1.62–2.57 | <0.001 |
| SEM excluding healthcare-provider recommendation (SP3) | Social processes → vaccination intention | Standardised β | 0.24 | 0.17–0.31 | <0.001 |
| SEM excluding healthcare-provider recommendation (SP3) | Practical accessibility → vaccination intention | Standardised β | 0.38 | 0.31–0.45 | <0.001 |

**Note:** aOR, adjusted odds ratio; β, standardised structural-equation coefficient; CI, confidence interval; SP3, healthcare-provider recommendation item; SEM, structural equation model. The site-clustered robust analysis re-estimated the fully adjusted primary logistic-regression model using healthcare-site-level cluster-robust standard errors, with the same covariate specification as primary Model 3. The SP3-excluded logistic model retained the same covariates as primary Model 3, except that the social-processes score was recalculated after excluding SP3. Reference categories for categorical predictors were male sex, age 18–29 years, junior middle school or below, monthly income <3000 yuan, rural residence, non-animal-related injury, clean minor wound, primary institution, emergency department, and formally verified previous tetanus immunisation. For the continuous BeSD construct scores, aORs represent the association per one-point increase in the corresponding 1–5 mean score. All reported P values were obtained directly from the fitted models.

Panel A2. Model-fit statistics for the sensitivity SEM excluding SP3 (n=657)

| **Model-fit statistic** | **Estimate** |
| --- | --- |
| χ² (df) | 266.68 (113) |
| P value | <0.001 |
| χ²/df | 2.360 |
| CFI | 0.951 |
| TLI | 0.943 |
| RMSEA | 0.045 |
| RMSEA 90% CI | 0.038–0.053 |
| SRMR | 0.044 |

**Note:** CFI, comparative fit index; TLI, Tucker–Lewis index; RMSEA, root mean square error of approximation; SRMR, standardised root mean square residual; CI, confidence interval.

**Panel B. Associations of prespecified BeSD constructs with alternative clinical outcomes in fully adjusted logistic-regression models (n=657)**

| **Outcome** | **Predictor** | **aOR** | **95% CI** | **P value** |
| --- | --- | --- | --- | --- |
| Documented active vaccination within 7 days | Thinking and feeling | 1.09 | 0.87–1.36 | 0.456 |
| Documented active vaccination within 7 days | Social processes | 1.24 | 0.98–1.58 | 0.076 |
| Documented active vaccination within 7 days | Practical accessibility | 1.41 | 1.10–1.81 | 0.007 |
| Algorithm-classified guideline-concordant prophylaxis | Thinking and feeling | 1.11 | 0.89–1.38 | 0.353 |
| Algorithm-classified guideline-concordant prophylaxis | Social processes | 1.32 | 1.04–1.68 | 0.024 |
| Algorithm-classified guideline-concordant prophylaxis | Practical accessibility | 1.53 | 1.17–2.01 | 0.002 |

**Note:** aOR, adjusted odds ratio; CI, confidence interval; BeSD, Behavioural and Social Drivers of Vaccination. Both alternative-outcome models adjusted for sex, age group, education, monthly income, residence, injury type, wound category, recruitment institution, recruitment setting, previous tetanus immunisation history, thinking and feeling, social processes, and practical accessibility. Reference categories for categorical covariates were male sex, age 18–29 years, junior middle school or below, monthly income <3000 yuan, rural residence, non-animal-related injury, clean minor wound, primary institution, emergency department, and formally verified previous tetanus immunisation. For the BeSD construct scores, aORs represent the association per one-point increase in the corresponding 1–5 mean score. Alternative-outcome models were exploratory because the temporal sequence between questionnaire completion, healthcare-provider recommendation, and documented clinical management could not be confirmed for all participants. Given the limited number of outcome events relative to the number of covariate parameters, estimates should be interpreted cautiously.
