# Supplementary material for: Behavioural and service-context correlates of tetanus vaccination intention after trauma exposure in China: a cross-sectional BeSD study
Source: Front Public Health. 2026 Sep 21;14:1941921. doi: 10.3389/fpubh.2026.1941921 (PMC13635121; doi:10.3389/fpubh.2026.1941921)
Supplement: Supplementary file 7 [file Table_6.docx]

Supplementary Table S6. Sensitivity analysis using the three-item vaccination-intention score as an alternative continuous outcome in the overall analytic cohort (n=657)

| **Predictor** | **Primary binary-outcome model, aOR (95% CI)** | **P value** | **Alternative continuous-outcome model, B (95% CI)** | **Standardised β** | **P value** |
| --- | --- | --- | --- | --- | --- |
| Female vs male | 1.10 (0.77–1.57) | 0.602 | 0.030 (−0.068 to 0.128) | 0.015 | 0.549 |
| Age 30–44 vs 18–29 years | 1.19 (0.72–1.95) | 0.497 | 0.040 (−0.097 to 0.177) | 0.018 | 0.568 |
| Age 45–59 vs 18–29 years | 0.98 (0.59–1.61) | 0.933 | −0.020 (−0.157 to 0.117) | −0.009 | 0.775 |
| Age ≥60 vs 18–29 years | 0.72 (0.41–1.26) | 0.256 | −0.100 (−0.257 to 0.057) | −0.038 | 0.211 |
| Senior high/technical vs junior middle or below | 0.98 (0.64–1.50) | 0.920 | 0.030 (−0.088 to 0.148) | 0.014 | 0.617 |
| College or above vs junior middle or below | 0.85 (0.53–1.35) | 0.492 | 0.010 (−0.127 to 0.147) | 0.004 | 0.886 |
| Income 3000–5999 vs <3000 yuan | 0.86 (0.57–1.30) | 0.472 | −0.040 (−0.158 to 0.078) | −0.018 | 0.505 |
| Income ≥6000 vs <3000 yuan | 0.84 (0.50–1.42) | 0.517 | −0.060 (−0.217 to 0.097) | −0.022 | 0.453 |
| Urban vs rural residence | 1.41 (0.97–2.05) | 0.073 | 0.080 (−0.018 to 0.178) | 0.037 | 0.110 |
| Animal-related vs non-animal-related injury | 1.69 (1.17–2.43) | 0.005 | 0.170 (0.052 to 0.288) | 0.081 | 0.005 |
| Contaminated/deep vs clean minor wound | 0.63 (0.41–0.96) | 0.030 | −0.110 (−0.208 to −0.012) | −0.055 | 0.028 |
| Severe/complex vs clean minor wound | 0.67 (0.39–1.14) | 0.139 | −0.090 (−0.227 to 0.047) | −0.039 | 0.199 |
| County-level or above hospital vs primary institution | 0.93 (0.65–1.34) | 0.711 | −0.030 (−0.148 to 0.088) | −0.014 | 0.617 |
| Surgical outpatient vs emergency department | 1.47 (0.89–2.43) | 0.128 | 0.070 (−0.067 to 0.207) | 0.025 | 0.317 |
| Animal-injury clinic vs emergency department | 1.21 (0.75–1.97) | 0.437 | 0.050 (−0.107 to 0.207) | 0.017 | 0.532 |
| Primary vaccination/wound-care clinic vs emergency department | 1.04 (0.62–1.73) | 0.883 | 0.020 (−0.137 to 0.177) | 0.006 | 0.803 |
| Self-reported inadequate history vs formally verified history | 1.30 (0.77–2.19) | 0.330 | 0.060 (−0.097 to 0.217) | 0.021 | 0.453 |
| Unknown/uncertain history vs formally verified history | 0.98 (0.64–1.51) | 0.942 | −0.010 (−0.128 to 0.108) | −0.004 | 0.868 |
| Thinking and feeling score | 1.46 (1.18–1.80) | <0.001 | 0.170 (0.111 to 0.229) | 0.154 | <0.001 |
| Social processes score | 1.68 (1.36–2.07) | <0.001 | 0.240 (0.181 to 0.299) | 0.221 | <0.001 |
| Practical accessibility score | 2.08 (1.66–2.62) | <0.001 | 0.330 (0.271 to 0.389) | 0.337 | <0.001 |

**Note:** The alternative continuous outcome was calculated as the mean of INT1–INT3, with higher scores indicating stronger vaccination intention. The primary binary-outcome estimates correspond to the fully adjusted logistic-regression Model 3. The alternative continuous-outcome model used multivariable linear regression with the same sociodemographic, trauma-related, healthcare-related, and BeSD predictors as Model 3. Reference categories were male sex, age 18–29 years, junior middle school or below, monthly income <3000 yuan, rural residence, non-animal-related injury, clean minor wound, primary institution, emergency department, and formally verified previous tetanus immunisation. For the BeSD construct scores, aORs represent the association per one-point increase in the corresponding 1–5 mean score. B represents the unstandardised linear-regression coefficient and β the standardised coefficient. Because the two models used different outcome scales and link functions, aORs and B coefficients should not be compared directly in magnitude; consistency was evaluated according to the direction and substantive pattern of associations. aOR, adjusted odds ratio; CI, confidence interval; BeSD, Behavioural and Social Drivers of Vaccination.
