# Supplementary material for: Behavioural and service-context correlates of tetanus vaccination intention after trauma exposure in China: a cross-sectional BeSD study
Source: Front Public Health. 2026 Sep 21;14:1941921. doi: 10.3389/fpubh.2026.1941921 (PMC13635121; doi:10.3389/fpubh.2026.1941921)
Supplement: Supplementary file 8 [file Table_7.docx]

**Supplementary Table S7. Stratified analysis of algorithm-classified guideline-concordant prophylaxis by immunisation-history classification in the overall analytic cohort (n=657)**

| **Analysis population** | **Total, n** | **Guideline-concordant prophylaxis, n** | **Proportion, %** | **95% CI** |
| --- | --- | --- | --- | --- |
| Overall analytic cohort | 657 | 119 | 18.1 | 15.36–21.24 |
| Formally verified previous immunisation information | 190 | 45 | 23.7 | 18.20–30.21 |
| Non-unknown questionnaire-based immunisation-history classification | 311 | 64 | 20.6 | 16.46–25.42 |
| Self-reported no vaccination or most recent dose >10 years previously | 121 | 19 | 15.7 | 10.29–23.23 |
| Unknown or uncertain immunisation history | 346 | 55 | 15.9 | 12.42–20.12 |

Note: The non-unknown immunisation-history classification comprised participants with formally verified previous vaccination and those in the self-reported inadequate-history category. Proportions are presented with Wilson 95% confidence intervals.
