# Supplementary material for: Behavioural and service-context correlates of tetanus vaccination intention after trauma exposure in China: a cross-sectional BeSD study
Source: Front Public Health. 2026 Sep 21;14:1941921. doi: 10.3389/fpubh.2026.1941921 (PMC13635121; doi:10.3389/fpubh.2026.1941921)
Supplement: Supplementary file 9 [file Table_8.docx]

**Supplementary Table S8. Association of injury type with documented active vaccination within 7 days in the overall analytic cohort (n=657)**

**Panel A. Association of injury type with documented active vaccination across models (n=657)**

| **Model** | **Animal-related vs non-animal-related injury** | **95% CI** | **P value** |
| --- | --- | --- | --- |
| Crude model | 2.63 | 1.76–3.93 | <0.001 |
| Model A | 1.94 | 1.22–3.08 | 0.005 |
| Model B | 1.72 | 1.06–2.79 | 0.028 |

**Panel B. Full estimates from Model B (n=657)**

| **Predictor** | **aOR** | **95% CI** | **P value** |
| --- | --- | --- | --- |
| Female vs male | 0.98 | 0.65–1.48 | 0.923 |
| Age 30–44 vs 18–29 years | 1.12 | 0.68–1.84 | 0.655 |
| Age 45–59 vs 18–29 years | 0.95 | 0.56–1.61 | 0.849 |
| Age ≥60 vs 18–29 years | 0.82 | 0.45–1.49 | 0.516 |
| Senior high/technical vs junior middle or below | 1.08 | 0.69–1.69 | 0.736 |
| College or above vs junior middle or below | 1.14 | 0.70–1.87 | 0.601 |
| Income 3000–5999 vs <3000 yuan | 1.05 | 0.70–1.58 | 0.814 |
| Income ≥6000 vs <3000 yuan | 1.18 | 0.72–1.94 | 0.513 |
| Urban vs rural residence | 1.10 | 0.75–1.61 | 0.625 |
| Animal-related vs non-animal-related injury | 1.72 | 1.06–2.79 | 0.028 |
| Contaminated/deep vs clean minor wound | 1.18 | 0.70–1.99 | 0.533 |
| Severe/complex vs clean minor wound | 1.31 | 0.71–2.42 | 0.386 |
| County-level or above hospital vs primary institution | 0.96 | 0.64–1.44 | 0.844 |
| Surgical outpatient vs emergency department | 0.82 | 0.47–1.43 | 0.486 |
| Animal-injury clinic vs emergency department | 1.47 | 0.86–2.53 | 0.161 |
| Primary vaccination/wound-care clinic vs emergency department | 1.25 | 0.71–2.20 | 0.438 |
| Self-reported inadequate history vs formally verified history | 1.16 | 0.62–2.17 | 0.645 |
| Unknown/uncertain history vs formally verified history | 0.81 | 0.48–1.36 | 0.425 |
| Thinking and feeling score | 1.09 | 0.87–1.36 | 0.456 |
| Social processes score | 1.24 | 0.98–1.58 | 0.076 |
| Practical accessibility score | 1.41 | 1.10–1.81 | 0.007 |

**Note:** The outcome was documented active tetanus vaccination within 7 days; absence of documented active vaccination within 7 days was the reference outcome. The crude model was unadjusted. Model A adjusted for sex, age group, education, monthly income, residence, wound category, recruitment institution, recruitment setting, and previous tetanus immunisation history. Model B additionally included thinking and feeling, social processes, and practical accessibility. Non-animal-related injury was the reference category for the exposure of interest. Other categorical reference groups were male sex, age 18–29 years, junior middle school or below, monthly income <3000 yuan, rural residence, clean minor wound, primary institution, emergency department, and formally verified previous tetanus immunisation. For the BeSD construct scores, aORs represent the association per one-point increase in the corresponding 1–5 mean score. OR, odds ratio; aOR, adjusted odds ratio; CI, confidence interval; BeSD, Behavioural and Social Drivers of Vaccination.
