# Supplementary material for: Behavioural and service-context correlates of tetanus vaccination intention after trauma exposure in China: a cross-sectional BeSD study
Source: Front Public Health. 2026 Sep 21;14:1941921. doi: 10.3389/fpubh.2026.1941921 (PMC13635121; doi:10.3389/fpubh.2026.1941921)
Supplement: Supplementary file 10 [file Table_9.docx]

**Supplementary Table S9 Factors associated with documented active tetanus vaccination within 7 days among clinically indicated participants reporting affirmative vaccination willingness (n = 333)**

| **Predictor** | **Transition Model 1 aOR (95% CI)** | **P value** | **Transition Model 2 aOR (95% CI)** | **P value** |
| --- | --- | --- | --- | --- |
| Female vs male | 0.92 (0.55–1.53) | 0.748 | 0.95 (0.56–1.61) | 0.849 |
| Age 30–44 vs 18–29 years | 1.10 (0.58–2.10) | 0.773 | 1.07 (0.55–2.08) | 0.842 |
| Age 45–59 vs 18–29 years | 0.95 (0.49–1.85) | 0.880 | 0.91 (0.46–1.81) | 0.788 |
| Age ≥60 vs 18–29 years | 0.78 (0.37–1.64) | 0.513 | 0.75 (0.35–1.61) | 0.461 |
| Senior high/technical vs junior middle or below | 1.18 (0.66–2.12) | 0.581 | 1.10 (0.60–2.02) | 0.758 |
| College or above vs junior middle or below | 1.42 (0.74–2.71) | 0.288 | 1.25 (0.64–2.43) | 0.512 |
| Income 3000–5999 vs <3000 yuan | 1.19 (0.67–2.10) | 0.549 | 1.12 (0.62–2.02) | 0.706 |
| Income ≥6000 vs <3000 yuan | 1.58 (0.81–3.08) | 0.179 | 1.33 (0.67–2.64) | 0.415 |
| Urban vs rural residence | 1.08 (0.66–1.76) | 0.758 | 1.05 (0.63–1.75) | 0.851 |
| Animal-related vs non-animal-related injury | 2.10 (1.20–3.67) | 0.009 | 1.84 (1.02–3.31) | 0.042 |
| Contaminated/deep vs clean minor wound | 0.92 (0.51–1.66) | 0.781 | 0.91 (0.50–1.67) | 0.761 |
| Severe/complex vs clean minor wound | 1.20 (0.60–2.38) | 0.602 | 1.09 (0.54–2.21) | 0.811 |
| County-level or above hospital vs primary institution | 1.07 (0.63–1.82) | 0.802 | 1.01 (0.58–1.75) | 0.972 |
| Surgical outpatient vs emergency department | 0.78 (0.39–1.58) | 0.492 | 0.82 (0.40–1.69) | 0.592 |
| Animal-injury clinic vs emergency department | 1.54 (0.76–3.12) | 0.231 | 1.36 (0.66–2.81) | 0.406 |
| Primary vaccination/wound-care clinic vs emergency department | 1.21 (0.60–2.45) | 0.596 | 1.16 (0.56–2.40) | 0.688 |
| Self-reported inadequate vs formally verified history | 1.28 (0.63–2.59) | 0.493 | 1.20 (0.58–2.48) | 0.622 |
| Unknown/uncertain vs formally verified history | 0.81 (0.44–1.49) | 0.497 | 0.78 (0.42–1.46) | 0.437 |
| Thinking and feeling score | — | — | 1.10 (0.80–1.51) | 0.551 |
| Social processes score | — | — | 1.29 (0.96–1.73) | 0.09 |
| Practical accessibility score | — | — | 1.62 (1.22–2.15) | <0.001 |

Note: The analysis was restricted to 333 participants who reported affirmative vaccination willingness and had a guideline-based indication for active tetanus vaccination; 100 had documented active vaccination within 7 days and 233 did not. Transition Model 1 adjusted for sex, age, education, monthly income, residence, injury type, wound category, recruitment institution, recruitment setting, and previous tetanus immunisation history. Transition Model 2 additionally included thinking and feeling, social processes, and practical accessibility scores. aOR, adjusted odds ratio; CI, confidence interval. Given the limited number of documented vaccination events, this analysis was considered exploratory. The reference outcome was no documented active vaccination within 7 days. Reference categories for categorical predictors were male sex, age 18–29 years, junior middle school or below, monthly income <3000 yuan, rural residence, non-animal-related injury, clean minor wound, primary institution, emergency department, and formally verified previous tetanus immunisation. For the BeSD construct scores, aORs represent the association per one-point increase in the corresponding 1–5 mean score.
